# Preservation of ALYREF Phase Separation Mitigates Doxorubicin‐Induced Cardiomyocyte DNA Damage and Cardiotoxicity

**DOI:** 10.1002/advs.202505270

**Published:** 2025-09-03

**Authors:** Xinlu Gao, Yifu Shen, Zhihui Xiao, Zhenbo Han, Xu Liu, Ao Cai, Yanan Tian, Guang Lian, Wenya Ma, Yining Liu, Rui Gong, Hanjing Li, Xiuxiu Wang, Zhongyu Ren, Naufal Zagidullin, Lei Yu, Ye Tian, Yu Liu, Zhenwei Pan, Baofeng Yang, Benzhi Cai

**Affiliations:** ^1^ Department of Pharmacy at the Second Affiliated Hospital Harbin Medical University Harbin 150086 China; ^2^ State Key Laboratory of Frigid Zone Cardiovascular Diseases (SKLFZCD) Department of Pharmacology (The State‐Province Key Laboratories of Biomedicine‐Pharmaceutics of China Key Laboratory of Cardiovascular Research Ministry of Education) College of Pharmacy Harbin Medical University Harbin 150081 China; ^3^ Department of Laboratory Medicine at The Fourth Affiliated Hospital Harbin Medical University Harbin 150023 China; ^4^ Department of Pharmacology and Regenerative Medicine University of Illinois College of Medicine Chicago IL 60612 USA; ^5^ Department of Pharmacy Xijing Hospital Fourth Military Medical University Xi'an Shanxi 710032 China; ^6^ Department of Internal Diseases Bashkir State Medical University Ufa 450008 Russia; ^7^ Department of Orthopedic Surgery The First Affiliated Hospital of Harbin Medical University Harbin 150001 China; ^8^ Department of Pathophysiology and the Key Laboratory of Cardiovascular Pathophysiology Harbin Medical University Harbin 150081 China; ^9^ Institute of Clinical Pharmacy NHC Key Laboratory of Cell Transplantation the Heilongjiang Key Laboratory of Drug Research Harbin Medical University Harbin 150081 China

**Keywords:** ALYREF, cardiotoxicity, DNA damage, doxorubicin, liquid–liquid phase separation

## Abstract

The clinical utility of the anticancer agent doxorubicin (DOX) is limited by its dose‐dependent cardiotoxicity. ALYREF, a nuclear protein that preserves genomic stability through interactions with intranuclear components or as an m⁵C‐binding regulator of mRNA maturation and export, has not been previously implicated in DOX‐induced cardiotoxicity (DIC). Here, the role and underlying mechanisms of ALYREF in the pathogenesis of DIC are investigated. The findings demonstrate that ALYREF expression is markedly reduced in a murine model of DIC. Myocardial‐specific overexpression of ALYREF attenuates DOX‐induced DNA damage and cardiomyocyte apoptosis, whereas cardiac‐specific knockout of ALYREF (ALYREF CKO) exacerbates DOX‐induced cardiac dysfunction. Mechanistically, it is identified that nuclear DOX directly binds to the aspartate residue (D171) within the intrinsically disordered regions (IDRs) of ALYREF, disrupting its liquid–liquid phase separation (LLPS) and promoting its ubiquitin‐mediated degradation. The condensate state of ALYREF is essential for maintaining the integrity of the NORAD‐activated ribonucleoprotein complex 1 (NARC1). Consequently, disruption of ALYREF LLPS leads to dissociation of the NARC1 complex, resulting in DNA damage and apoptosis in CMs. Collectively, these findings reveal a previously unrecognized mechanism by which DIC via interference with ALYREF condensates, offering new insight into the molecular basis of DIC.

## Graphical abstract

Binding of Doxorubicin to ALYREF disrupts its phase‐separated condensate and induces DNA damage and apoptosis in cardiomyocytes

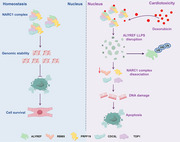

## 1 Introduction

DOX is an anthracycline‐based chemotherapeutic agent widely used in the treatment of both solid and hematologic malignancies.^[^ ^1^ ^]^ Despite its clinical efficacy, DOX administration is associated with dose‐dependent cardiotoxicity, manifesting as arrhythmias, irreversible ventricular dysfunction, and ultimately heart failure.^[^ ^2^, ^3^ ^]^ In cancer patients, DIC has emerged as a leading cause of nontumor‐related mortality. Although dexrazoxane is currently the only FDA‐approved cardioprotective agent for mitigating DIC, its use is limited by concerns regarding oncologic outcomes. Thus, identifying novel therapeutic targets and elucidating the molecular basis of DIC remain pressing clinical needs.

The pathogenesis of DIC is multifactorial. Previous studies have primarily focused on cytoplasmic mechanisms, including mitochondrial dysfunction, oxidative stress, impaired autophagy, and inflammation.^[^ ^4^, ^5^, ^6^, ^7^, ^8^, ^9^, ^10^ ^]^ However, DOX is also capable of nuclear entry, where it forms ternary complexes with DNA and topoisomerase IIβ (TOP IIβ), resulting in DNA double‐strand breaks.^[^ ^9^ ^]^ These findings underscore the importance of nuclear DNA damage in the development of DIC and suggest that mitigating such damage may be a viable therapeutic strategy.

Identification of molecular targets that regulate DOX‐induced DNA damage in CMs is crucial for advancing DIC therapy. ALYREF (Aly/REF export factor) is a nuclear RNA‐binding protein with m⁵C‐reading capacity that facilitates RNA maturation and export.^[^ ^11^, ^12^, ^13^, ^14^ ^]^ Beyond its role in RNA metabolism, ALYREF contributes to genomic stability as a component of the NARC1.^[^ ^15^ ^]^ It also acts as a coregulator of R‐loop homeostasis by binding R‐loops and promoting UAP56‐mediated R‐loop resolution during transcription.^[^ ^16^ ^]^ Although ALYREF has been implicated in tumorigenesis via regulation of m⁵C‐modified transcripts, its potential role in protecting CMs from DOX‐induced DNA damage remains unclear.

LLPS enables the formation of membraneless intracellular condensates through multivalent, weak interactions among biomolecules, and is typically driven by features such as IDRs, hydrophobic residues, and charged domains.^[^ ^17^, ^18^, ^19^, ^20^ ^]^ Aberrant LLPS has been implicated in numerous pathologies, including neurodegenerative and cardiovascular diseases.^[^ ^21^, ^22^, ^23^, ^24^ ^]^ For instance, altered LLPS of HIP‐55 via phosphorylation has been shown to protect against adrenergic‐induced heart failure, highlighting a regulatory role for LLPS in cardiac function.^[^ ^25^ ^]^ In this study, we identify ALYREF as a key molecular target in DIC. Cardiac‐specific overexpression of ALYREF mitigated DOX‐induced DNA damage, apoptosis, and cardiac dysfunction in mice. Through molecular dynamics simulations and cellular thermal shift assays, we show that DOX binds directly and specifically to the aspartate residue (D171) within the IDRs of ALYREF, disrupting its phase separation properties and promoting disassembly of the NARC1 complex. The resulting loss of NARC1 integrity leads to CM DNA damage and apoptosis, ultimately impairing cardiac function. Notably, overexpression of a mutant ALYREF that retains m⁵C‐binding capacity improved cardiac contractility in DOX‐treated tumor‐bearing animals without promoting tumorigenesis. These findings establish a mechanistic link between disrupted ALYREF phase separation and DIC, and suggest that modulating ALYREF function may represent a novel therapeutic approach for cardio protection in cancer patients.

## 2 Results

### 2.1 ALYREF Overexpression Attenuates Doxorubicin‐Induced DNA Damage and Apoptosis in Cardiomyocytes

**Figure 1:**
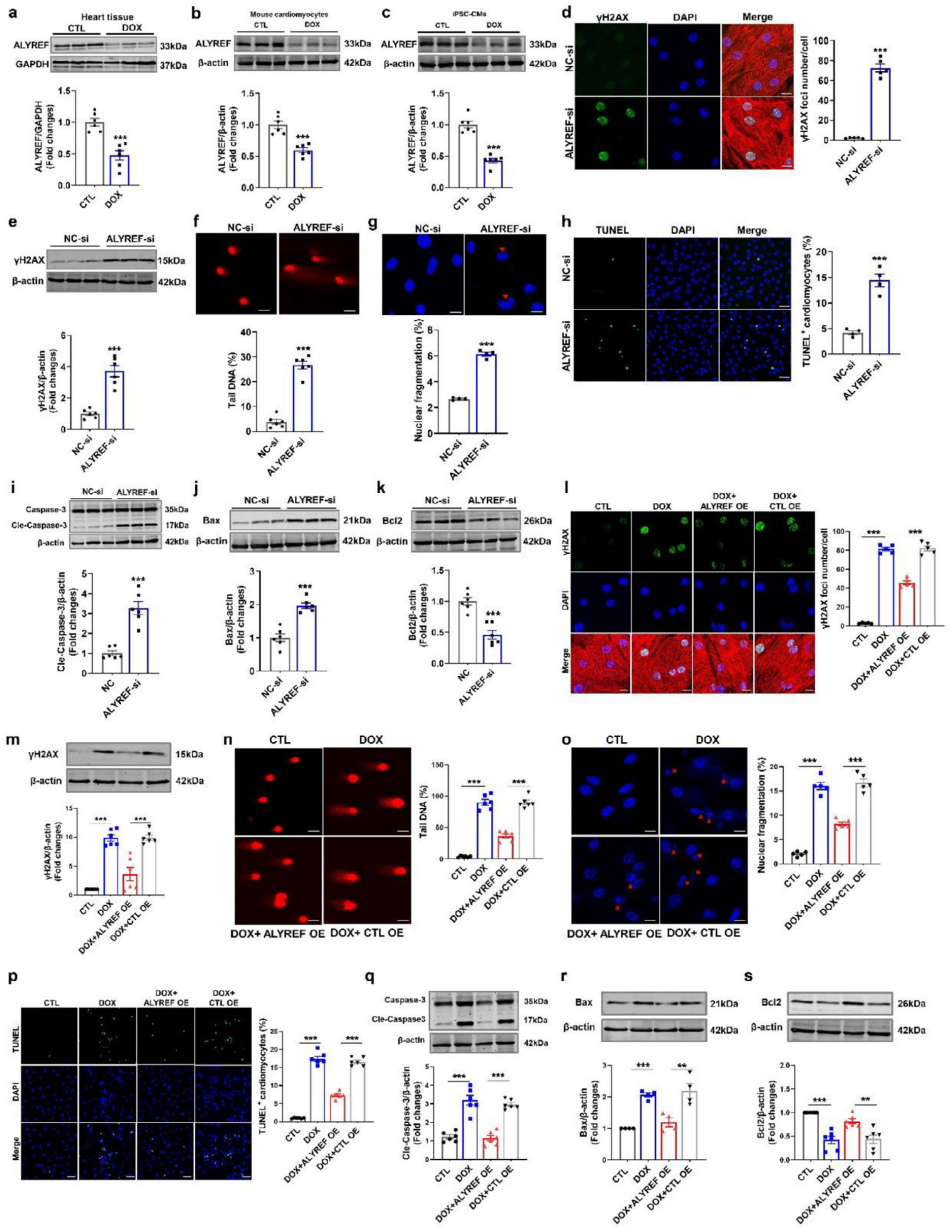
ALYREF regulates DNA damage and apoptosis in cardiomyocytes. a) Western blot analysis of ALYREF protein levels in DIC mouse hearts (*n* = 6). b,c) Western blot analysis of ALYREF protein levels in DOX‐treated CMs (*n* = 6) or hiPSC‐CMs (*n* = 6). d–k) CMs transfected with ALYREF siRNA or negative control (NC). (d) Representative images (left) and quantification (right) of immunostaining of the DNA damage marker γH2AX (green) in CMs (*n* = 5). CMs were counterstained with α‐actinin (red, a cardiac marker) and DAPI (blue). Scale bar = 50 µm. (e) Western blot analysis of γH2AX protein levels in CMs (*n*=6). (f) Representative images and quantification of comet assay in CMs (*n* = 6). Scale bar = 50 µm. (g) Representative images and quantification of immunostaining of the DAPI in CMs (*n* = 4). Scale bar=50 µm. (h) Representative images and quantification of TUNEL staining in CMs (*n* = 4). Scale bar = 50 µm. (i–k) Western blot analysis of Cleaved‐Caspase‐3 (*n* = 6), Bax (*n* = 6), and Bcl2 (*n* = 6) protein levels in CMs. l–s) CMs transfected with ALYREF plasmid and then treated with DOX. (l) Representative images and quantification of immunostaining of γH2AX (green), α‐actinin (red, a cardiac marker) and DAPI (blue) in CMs (*n* = 5). Scale bar = 50 µm. (m) Western blot analysis of γH2AX protein levels in CMs (*n* = 6). (n) Representative images and quantification of comet assay in CMs (*n* = 6). Scale bar=50 µm. (o) Representative images and quantification of immunostaining of the DAPI in CMs (*n* = 5). Scale bar = 50 µm. (p) Representative images and quantification of TUNEL staining in CMs (*n* = 6). Scale bar = 50 µm. (q–s) Western blot analysis of Cleaved‐Caspase‐3 (*n*=6), Bax (*n*=4), and Bcl2 (*n* = 6) protein levels in CMs. Data are presented as mean ± SEM. Statistical analysis was performed by (a–k) Student's *t*‐test, and (l–s) by one‐way ANOVA with Tukey's multiple comparisons test. ^**^ *p* < 0.01, and ^***^ *p* < 0.001.

To explore the role of ALYREF in DIC, we assessed its expression under DOX exposure both in vivo and in vitro. ALYREF expression was significantly reduced in DOX‐treated mouse hearts, mouse CMs, and human induced pluripotent stem cell‐derived cardiomyocytes (hiPSC‐CMs) (**Figure** **1**a–c). In contrast, ALYREF expression in mouse cardiac fibroblasts and endothelial cells remained unchanged following DOX treatment (Figure S1a, Supporting Information).

Given the strong association between DIC and both DNA damage and apoptosis.^[^ ^10^, ^26^ ^]^ we next evaluated the consequences of ALYREF knockdown in CMs. Immunofluorescence analysis revealed that ALYREF silencing markedly increased the number of γH2AX foci, an indicator of DNA damage, compared with negative control (NC) CMs (Figure 1d; Figure S1b, Supporting Information). Consistently, immunoblotting demonstrated elevated γH2AX protein levels following ALYREF knockdown (Figure 1e). ALYREF‐deficient CMs also exhibited enhanced genomic instability, as evidenced by increased comet tail DNA percentage and nuclear fragmentation (Figure 1f,g). Apoptotic cells were elevated, as shown by increased TdT‐mediated dUTP nick‐end labeling (TUNEL)‐positive cells and a higher proportion of apoptotic cells detected by flow cytometry (Figure 1h; Figure S1c, Supporting Information). Western blot analysis further confirmed increased expression of proapoptotic proteins (Cle‐Caspase‐3 and Bax) and decreased expression of the antiapoptotic protein Bcl2 (Figure 1i–k; Figure S1d, Supporting Information). These findings indicate that ALYREF contributes to genomic stability in CMs, and its depletion promotes DNA damage and apoptosis.

Since DOX downregulates ALYREF in CMs (Figure 1a–c), we next examined whether overexpression of ALYREF would be protective against DOX‐induced injury (Figure S1e, Supporting Information). DOX treatment markedly increased γH2AX foci and protein levels in CMs, both of which were attenuated by ALYREF overexpression (Figure 1l,m). Moreover, ALYREF overexpression reduced comet tail DNA and nuclear fragmentation in DOX‐exposed CMs (Figure 1n,o). Forced expression of ALYREF also diminished DOX‐induced apoptosis, as shown by TUNEL staining, flow cytometry, and modulation of apoptosis‐related protein levels (Figure 1p–s; Figure S1f,g, Supporting Information). Collectively, these data demonstrate that ALYREF overexpression attenuates DOX‐induced DNA damage and apoptosis in CMs.

### 2.2 Cardiac‐Specific ALYREF Deficiency Exacerbates Doxorubicin‐Induced Cardiotoxicity in Mice

**Figure 2:**
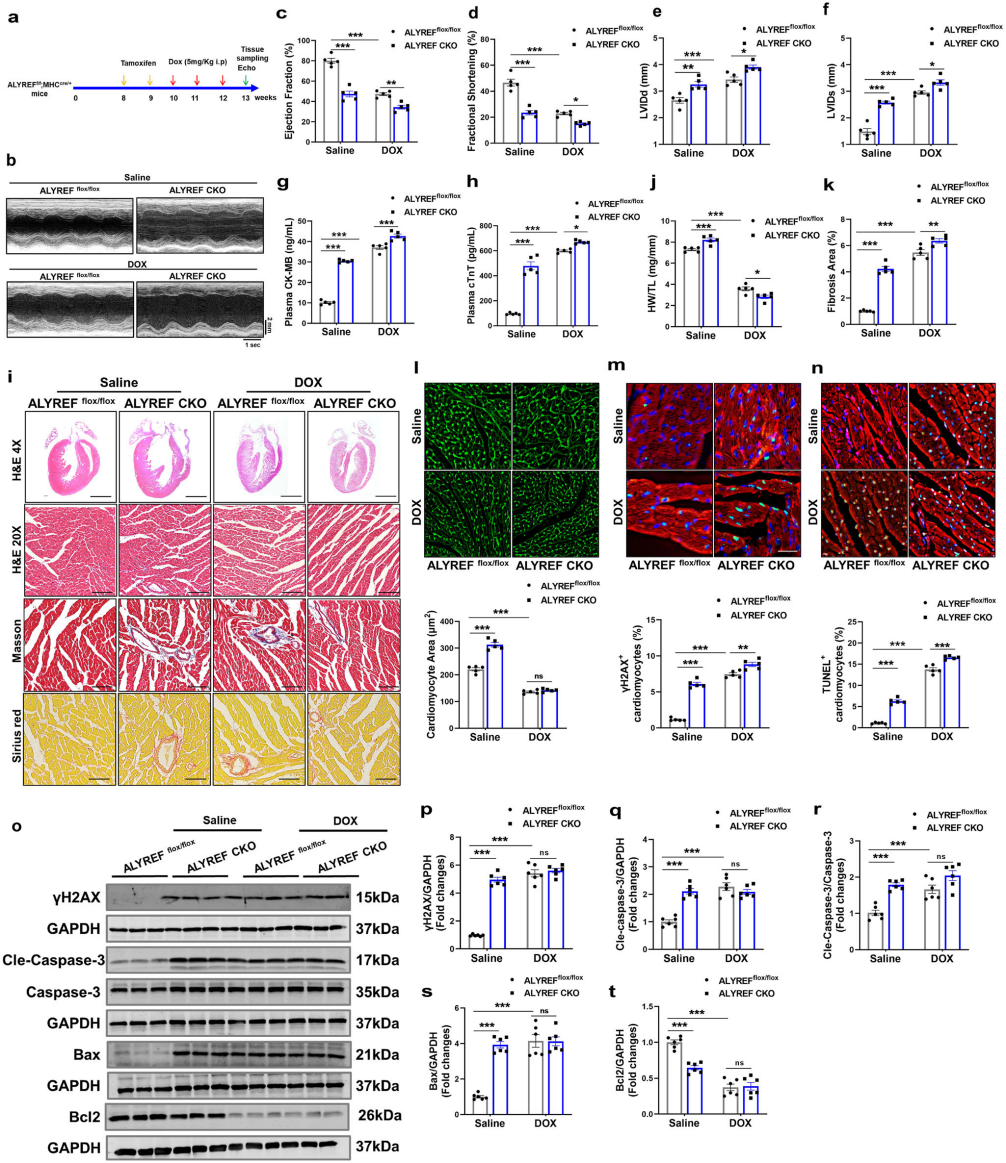
Cardiac‐specific ALYREF deficiency exacerbates doxorubicin‐induced cardiotoxicity in mice. a) Schematic diagram of the experimental procedure for constructing ALYREF CKO mice for DIC.Adult mice received continuous intraperitoneal injections of tamoxifen (30 mg/kg/day) for 5 days to induce ALYREF gene knockout in the heart at week 8. After 2 weeks, a DIC model was induced. b–f) Cardiac function was examined by echocardiography in ALYREF CKO or ALYREF^flox/flox^ mice treated with DOX or saline. Quantitative analysis of ejection fraction (EF), shortening fraction (FS), end‐diastolic left ventricular internal diameter (LVIDd), and end‐systolic left ventricular internal diameter (LVIDs) (*n* = 5). g,h) ELISA to measure creatine kinase‐MB (CK‐MB) and cardiac troponin T(cTnT) levels in the serum (*n* = 5). i) Representative hematoxylin and eosin (H&E), Masson, and Sirius red staining of heart (*n* = 5). j) Heart weight to tibia length (HW/TL) ratio (*n* = 5). k) Quantitative analysis of Masson staining (*n* = 5). l) Representative and quantitative analysis of WGA staining (*n* = 5). m) Representative images and quantification of immunostaining of γH2AX (green) in heart (*n* = 5). Cardiac tissue was counterstained with α‐actinin (red, a cardiac marker) and DAPI (blue). Scale bar = 50 µm. n) Representative images and quantification of TUNEL staining in heart (*n* = 5). Scale bar = 50 µm. o–t) Western blot analysis of γH2AX (*n* = 6), Cle‐Caspase‐3 (*n* = 6), Bax (*n* = 6), and Bcl2 (*n* = 6) protein levels in heart. Data are presented as mean ± SEM. (c–h,j–t) One‐way ANOVA with Tukey's multiple comparisons test was used. ^*^ *p* < 0.05, ^**^ *p* < 0.01, and ^***^ *p* < 0.001.

To investigate the functional role of ALYREF in the heart in vivo, we generated cardiac‐specific ALYREF knockout mice (ALYREF CKO) by crossing α‐myosin heavy chain (α‐MHC)‐Cre mice with ALYREF^flox/flox^ mice (Figure S2a,b, Supporting Information). Western blot analysis confirmed an ≈80% reduction in ALYREF protein levels in the hearts of ALYREF CKO mice compared with ALYREF^flox/flox^ mice (Figure S2c, Supporting Information), while ALYREF expression remained unaltered in noncardiac tissues (Figure S2d–g, Supporting Information), suggesting that the ALYREF CKO mice were successfully constructed. To assess the role of ALYREF in DIC model, ALYREF^flox/flox^ and ALYREF CKO mice were administered DOX intraperitoneally (**Figure** **2**a). Due to the high mortality observed in ALYREF CKO mice after 4 weeks of DOX treatment (Figure S2h, Supporting Information), we chose to analyze subsequent experiments after 3 weeks of DOX administration to mice. Echocardiography revealed that ALYREF CKO mice exhibited significant cardiac dysfunction, characterized by reduced left ventricular ejection fraction (EF) and fractional shortening (FS) (Figure 2b–d), as well as increased left ventricular internal diameters at end‐diastole (LVIDd) and end‐systole (LVIDs) (Figure 2e,f). Serum levels of creatine kinase‐MB (CK‐MB) and cardiac troponin T (cTnT), markers of myocardial injury, were elevated in ALYREF CKO mice relative to ALYREF^flox/flox^ controls (Figure 2g,h). Histological analysis by hematoxylin and eosin (H&E) staining showed enlarged left ventricular chambers and pathological cardiac hypertrophy in ALYREF‐deficient mice (Figure 2i), accompanied by an increased heart weight‐to‐tibial length ratio (HW/TL) (Figure 2j). Sirius red and Masson's trichrome staining revealed increased myocardial fibrosis, while wheat germ agglutinin (WGA) staining indicated hypertrophy of CMs (Figure 2i–l). Immunofluorescence analysis further demonstrated that ALYREF deficiency significantly increased the proportion of γH2AX and TUNEL‐positive CMs (Figure 2m,n). Consistent with these findings, Western blot confirmed elevated expression of γH2AX and proapoptotic proteins, along with reduced levels of antiapoptotic proteins (Figure 2o–t). Collectively, these results indicate that cardiac‐specific deletion of ALYREF induces DNA damage and apoptosis, culminating in myocardial dilation and impaired contractile function. Notably, DOX treatment further aggravated these pathological features in ALYREF CKO mice, as evidenced by myocardial contractile dysfunction, elevated serum CK‐MB and cTnT levels, increased fibrosis, and heightened cardiomyocyte DNA damage and apoptosis (Figure 2b–t). Taken together, our data underscore the essential role of ALYREF in preserving myocardial genomic integrity and contractile function during DIC.

### 2.3 Cardiac‐Specific Overexpression of ALYREF Attenuates Doxorubicin‐Induced Cardiotoxic Injury in Mice

**Figure 3:**
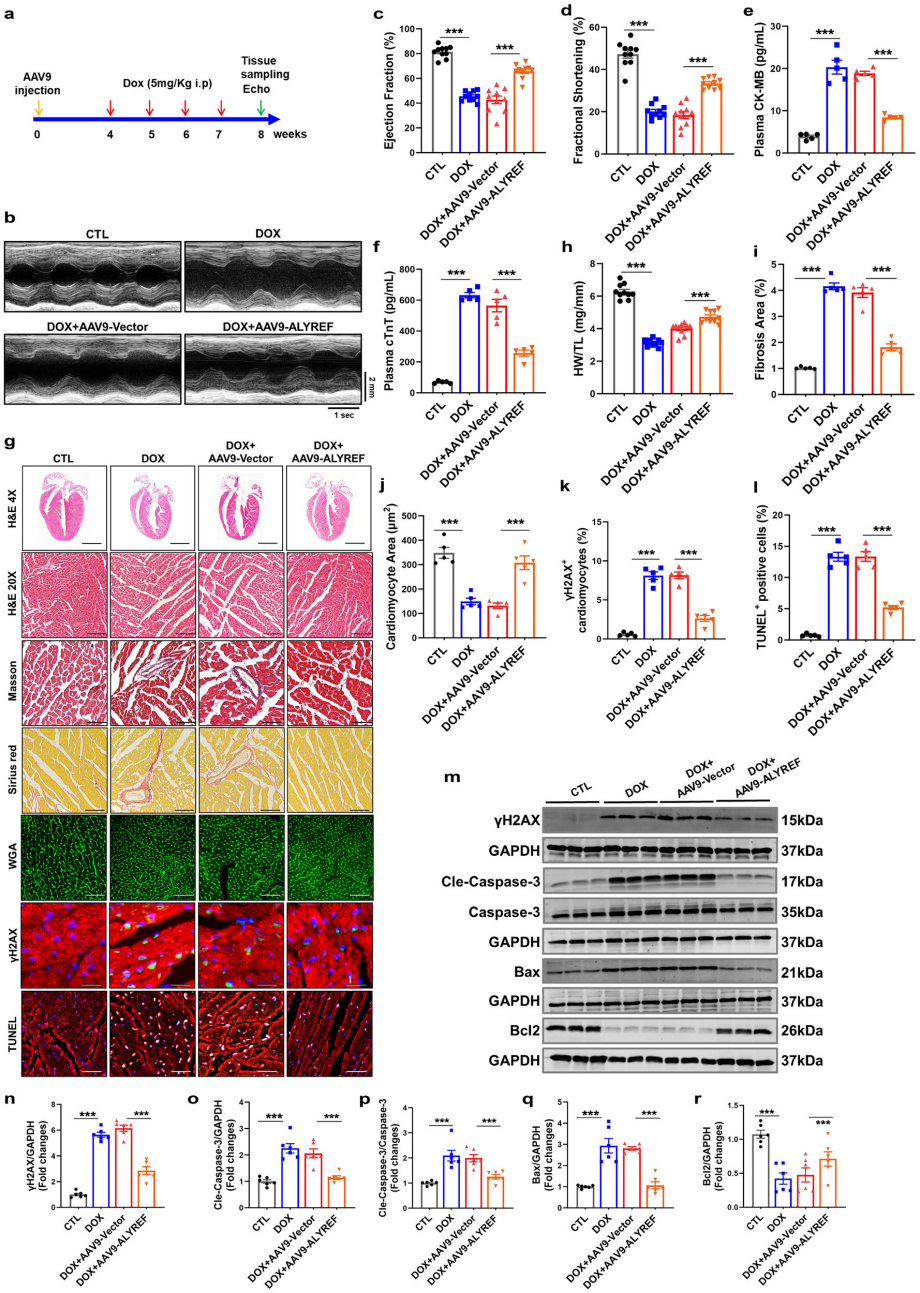
Cardiac‐specific overexpression of ALYREF attenuates doxorubicin‐induced cardiotoxicity. a) Schematic representation of the cardiac‐specific overexpression of ALYREF mice and DOX administration. The time points in the picture are representative. b–d) Cardiac function was examined by echocardiography in AAV9‐ALYREF CKO or AAV9‐Vector mice treated with DOX or saline. Quantitative analysis of EF, and FS (*n* = 10). e,f) ELISA to measure CK‐MB and cTnT levels in the serum (*n* = 5). g) Representative hematoxylin and eosin (H&E), Masson, Sirius red, WGA, γH2AX, and TUNEL staining of heart (*n* = 5). Cardiac tissue was counterstained with α‐actinin (red, a cardiac marker) and DAPI (blue). Scale bar = 50 µm. h) Heart weight to tibia length (HW/TL) ratio (*n* = 10). i–l) Quantitative analysis of Masson, WGA, γH2AX, and TUNEL staining (*n* = 5). m–r) Western blot analysis of γH2AX (*n* = 6), Cleaved‐Caspase‐3 (*n* = 6), Bax (*n* = 6), and Bcl2 (*n*= 6) protein levels in heart. Data are presented as mean ± SEM. (c–f, h–r) Statistical analysis was performed by one‐way ANOVA with Tukey's multiple comparisons test. ^**^ *p* < 0.01 and ^***^ *p* < 0.001.

To determine whether ALYREF confers protection against DOX‐induced myocardial injury in vivo, we generated mice with cardiac‐specific overexpression of ALYREF by administering recombinant adeno‐associated virus serotype 9 (AAV9) vectors carrying ALYREF under the control of the cTnT promoter via tail vein injection, prior to DOX exposure (**Figure** **3**a). Western blot analysis revealed that ALYREF protein levels in the myocardium were increased by ≈50% in AAV9‐ALYREF–treated mice compared with AAV9‐Vector controls (Figure S3a, Supporting Information). Immunofluorescence staining also confirmed enhanced ALYREF expression in myocardial tissue (Figure S3b, Supporting Information). Importantly, ALYREF expression in noncardiac tissues (liver, spleen, lung, and kidney) did not differ significantly between groups, confirming the successful construction of myocardium‐specific overexpressing ALYREF mice (Figure S3c–f, Supporting Information). Baseline cardiac function, assessed by echocardiography 4 weeks after AAV9 injection and prior to DOX administration, showed no significant differences between groups (Figure S3g,h, Supporting Information). Following DOX treatment, mice with ALYREF overexpression exhibited improved survival and preserved body weight compared with controls (Figure S3i,j, Supporting Information). Echocardiographic analysis demonstrated that cardiac‐specific overexpression of ALYREF significantly preserved left ventricular function relative to the DOX + AAV9‐Vector group (Figure 3b–d). In line with these findings, serum levels of cardiac injury markers, CK‐MB and cTnT, were significantly lower in the DOX + AAV9‐ALYREF group (Figure 3e,f). Histological analyses further showed that ALYREF overexpression mitigated DOX‐induced myocardial atrophy and fibrosis, as evidenced by H&E, Masson's trichrome, Sirius red, and WGA staining (Figure 3g–j). In addition, ALYREF overexpression reduced myocardial DNA damage and apoptosis, as assessed by γH2AX immunostaining, TUNEL staining, and apoptotic marker expression (Figure 3g,k–r). Collectively, these findings indicate that myocardial‐specific overexpression of ALYREF against DIC in mice.

### 2.4 ALYREF Regulates Doxorubicin‐Induced Cardiotoxicity Independently of its m^5^C Reader Function

**Figure 4:**
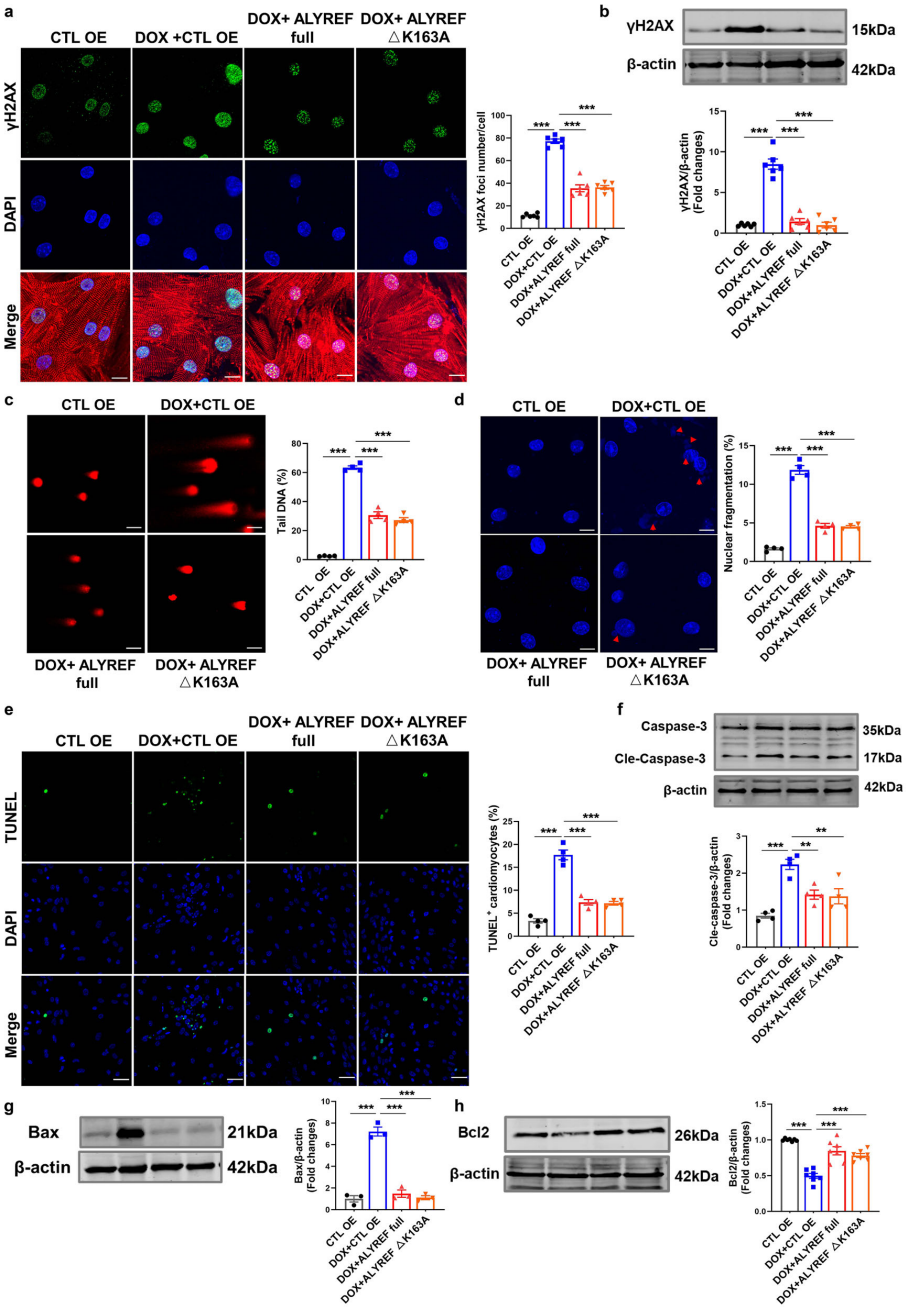
ALYREF regulates doxorubicin‐induced DNA damage and apoptosis in cardiomyocytes independent of m^5^C reader function. CMs transfected with ALYREF or ALYREF △K163A plasmid and then treated with DOX. a) Representative images and quantification of immunostaining of γH2AX (green), α‐actinin (red, a cardiac marker) and DAPI (blue) in CMs (*n* = 6). Scale bar = 50 µm. b) Western blot analysis of γH2AX protein levels in CMs (*n* = 6). c) Representative images and quantification of comet assay in CMs (*n* = 4). Scale bar = 50 µm. d) Representative images and quantification of immunostaining of the DAPI in CMs (*n* = 4). Scale bar = 50 µm. e) Representative images and quantification of TUNEL staining in CMs (*n* = 4). Scale bar = 50 µm. f–h) Western blot analysis of Cleaved‐Caspase‐3 (*n* = 4), Bax (*n* = 3), and Bcl2 (*n* = 7) protein levels in CMs. Data are presented as mean ± SEM. (a–h) Statistical analysis was performed by one‐way ANOVA with Tukey's multiple comparisons test. **p < 0.01, ^***^ *p* < 0.001.

ALYREF, an m⁵C‐binding protein, is known to participate in mRNA splicing, maturation, and nuclear export, thereby contributing to multiple cellular processes.^[^ ^27^ ^]^ To determine whether the role of ALYREF in DIC is dependent on its m⁵C reader function, we constructed a murine‐derived ALYREF K163 site mutant plasmid (ALYREF ΔK163A), and homologous sequence comparisons showed that this site is consistent with the human‐derived ALYREF m^5^C site (Figure S4a, Supporting Information). Unexpectedly, overexpression of the ALYREF ΔK163A in CMs reduced DOX‐induced γH2AX foci and protein levels to a similar extent as overexpression of wild‐type ALYREF (ALYREF full) (**Figure** **4**a,b). This was accompanied by reduced comet tail DNA percentage and decreased nuclear fragmentation (Figure 4c,d). Furthermore, DOX‐induced CM apoptosis was inhibited following ALYREF ΔK163A overexpression, as demonstrated by TUNEL staining, flow cytometry, and protein analyses (Figure 4e–h; Figure S4b,c, Supporting Information).Collectively, these findings indicate that ALYREF attenuates DOX‐induced DNA damage and apoptosis in CMs through an otherwise unknown functional mechanism.

### 2.5 Doxorubicin Disrupts the Liquid–Liquid Phase Separation of ALYREF

**Figure 5:**
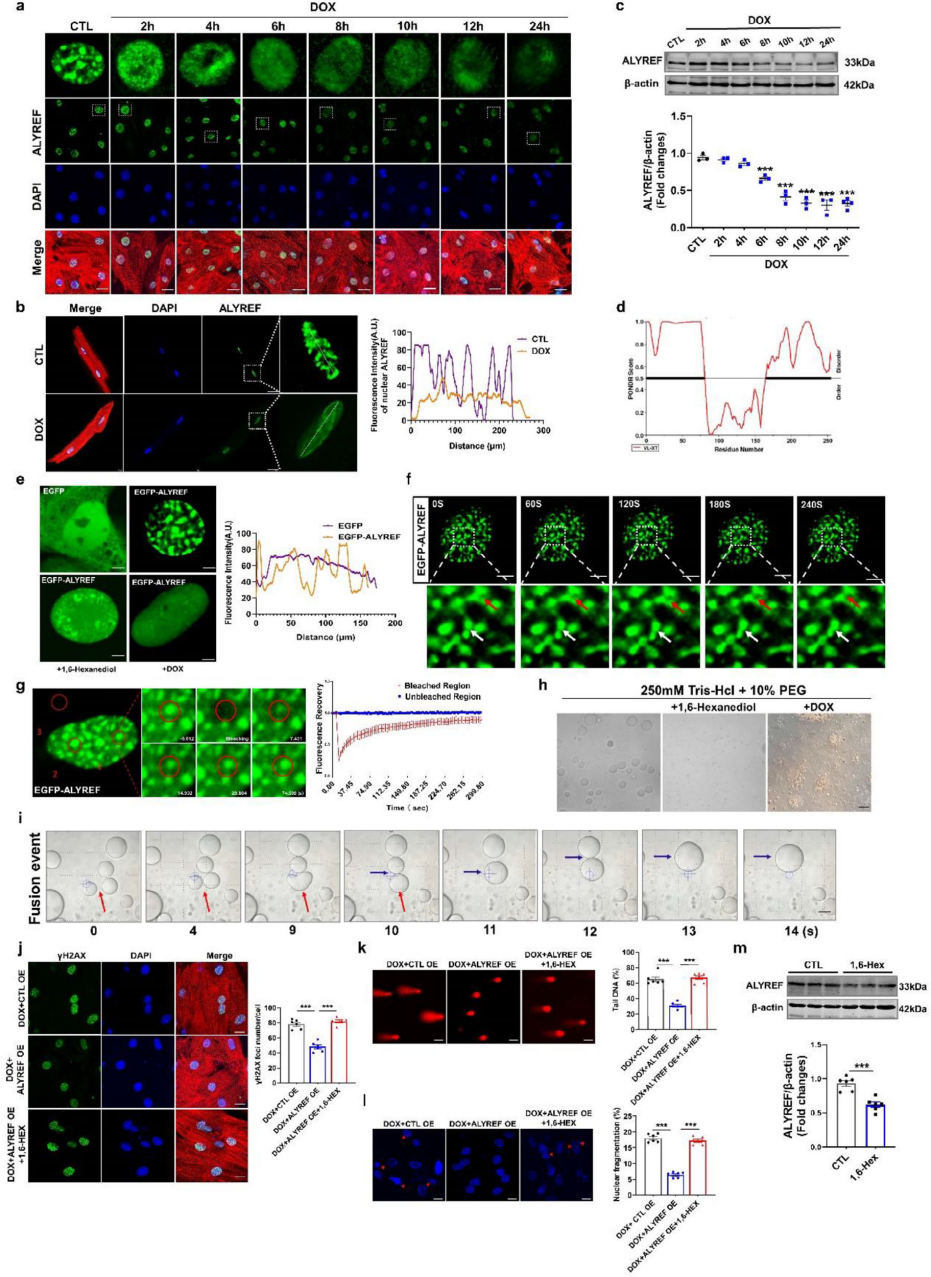
Doxorubicin induces DNA damage by disrupting the ALYREF liquid–liquid phase separation condensate in cardiomyocytes. a) Representative images of ALYREF (green) immunostaining in CMs treated with DOX at different time points (*n* = 4). CMs was counterstained with α‐actinin (red, a cardiac marker) and DAPI (blue). Scale bar = 50 µm. b) Representative images of ALYREF (green) immunostaining in CMs isolated from DIC mice (*n* = 3). c) Western blot analysis of ALYREF protein levels in CMs treated with DOX at different time points (*n* = 3). d) PONDR Predicts Disordered Regions (IDRs) in the Mouse ALYREF Protein Sequence. e) Representative images and quantification of fluorescence intensity of the CMs overexpressing either EGFP or EGFP‐ALYREF and then EGFP‐ALYREF treated with 5% 1,6‐hexanediol (1,6‐HEX) or DOX. Scale bars = 10 µm. f) Live‐cell time‐lapse imaging shows two adjacent EGFP‐ALYREF aggregates fused (white arrows) and dividing (red arrows) in CMs. Scale bar = 2.5 µm. g) Live‐cell images of fluorescence recovery after photobleaching (FRAP) experiments in CMs overexpressing EGFP‐ALYREF. Left) Representative images of FRAP experiments. (1) The region of photobleaching and analysis. (2) Positive control, un‐photobleached area and analysis. (3) Negative control, the region of un‐photobleaching. Right) Quantification of fluorescence intensity during FRAP assay. Scale bars = 5 µm and 2.5 µm (zoomed‐in image). h) Formation of ALYREF droplets in 250 mmol L^−1^ Tris‐Hcl and 10% PEG‐8000 solution, and then treated with 1,6‐HEX or DOX. Scale bars = 50 µm. i) The time‐lapse images display fusion of ALYREF droplets in vitro. Scale bars = 50 µm. j–l) CMs transfected with ALYREF and then treated with 1,6‐HEX (0.25%) or DOX. (j) Representative images and quantification of immunostaining of γH2AX (green), α‐actinin (red, a cardiac marker), and DAPI (blue) in CMs (*n* = 6). Scale bar = 50 µm. (k) Representative images and quantification of comet assay in CMs (*n* = 6). Scale bar = 50 µm. (l) Representative images and quantification of immunostaining of the DAPI in CMs (*n* = 6). Scale bar = 50 µm. m) Western blot analysis of ALYREF protein levels in CMs after treated with 1,6‐HEX (0.25%) (*n* = 6). Data are presented as mean ±SEM. Statistical analysis was performed by (c–m) Student's *t*‐test, and (j–l) by one‐way ANOVA with Tukey's multiple comparisons test. ^***^ *p* < 0.001.

In addition to altering ALYREF expression levels, DOX treatment affected its subnuclear localization in CMs. Following DOX exposure, ALYREF lost its punctate nuclear organization and instead became diffusely distributed throughout the nucleoplasm, without evidence of nuclear translocation (**Figure** **5**a). Similar diffuse nuclear localization of ALYREF was observed in CMs isolated from DIC mice (Figure 5b). Notably, this redistribution occurred as early as 2 h after DOX treatment, despite unaltered total protein levels, suggesting that ALYREF localization may influence its functional availability (Figure 5c). LLPS is a biophysical process by which intracellular biomolecules form membrane‐less, droplet‐like condensates via weak, multivalent interactions. Under physiological conditions, ALYREF displayed punctate nuclear structures resembling LLPS condensates. IDRs, which lack stable secondary structure and are typically enriched in low‐complexity sequences, are key drivers of LLPS in proteins.^[^ ^28^ ^]^ Bioinformatic analysis using the LLPS predictor PONDR revealed IDRs at both the N‐ and C‐terminal of ALYREF (Figure 5d), consistent with its LLPS potential. To experimentally assess the LLPS behavior of ALYREF, we generated an EGFP‐tagged ALYREF plasmid construct (EGFP‐ALYREF) and expressed it in CMs. Live‐cell imaging demonstrated that, unlike control EGFP, which showed diffuse cytoplasmic and nuclear distribution, EGFP‐ALYREF formed discrete nuclear condensates (Figure 5e, top). Treatment with 1,6‐hexanediol (1,6‐HEX), a chemical disruptor of phase‐separated condensates,^[^ ^29^ ^]^ or with DOX abolished these droplet‐like structures (Figure 5e, bottom). Time‐lapse imaging revealed that EGFP‐ALYREF condensates exhibited hallmark LLPS dynamics, including spontaneous fusion and fission events (Figure 5f). Fluorescence recovery after photobleaching (FRAP) further confirmed the liquid‐like behavior of ALYREF condensates, with fluorescence recovery occurring within 5 min of photobleaching (Figure 5g). These observations collectively indicate that ALYREF forms dynamic, droplet‐like condensates within the nuclei of CMs.

To validate these findings in vitro, we purified recombinant ALYREF protein and observed spherical droplet formation (Figure 5h, left). These condensates were sensitive to 1,6‐HEX treatment and were also disrupted by DOX, which caused droplet disintegration and structural irregularity (Figure 5h, middle and right). ALYREF droplets also displayed spontaneous fusion behavior when in contact (Figure 5i), reinforcing its intrinsic LLPS capability. To determine whether LLPS is functionally required for ALYREF‐mediated cardio protection, we overexpressed ALYREF in DOX‐treated CMs and cotreated with 1,6‐HEX. Disruption of LLPS by 1,6‐HEX abrogated the protective effect of ALYREF overexpression, as indicated by increased γH2AX foci, greater comet tail DNA percentage, and higher nuclear fragmentation (Figure 5j–l). These results suggest that the LLPS capacity of ALYREF is essential for limiting DOX‐induced DNA damage.

Our previous observations showed that DOX disrupts ALYREF condensates prior to downregulating ALYREF protein expression (Figure 5a,b). Similarly, treatment with 1,6‐HEX induced disorganization of ALYREF condensates, followed by reduced protein levels (Figure 5m), suggests a link between LLPS integrity and protein stability. To explore the mechanism underlying ALYREF downregulation, we treated CMs with the protein synthesis inhibitor cycloheximide (CHX), the proteasome inhibitor MG‐132, and the autophagy–lysosome inhibitor chloroquine (CQ). Western blot results showed that only MG‐132 prevented DOX‐induced ALYREF degradation, indicating a proteasome‐dependent mechanism (Figure S5a–c, Supporting Information). Additionally, DOX enhanced K48‐linked ubiquitination of ALYREF in CMs (Figure S5d, Supporting Information), implicating the ubiquitin–proteasome pathway in its degradation. Mass spectrometry identified E3 ubiquitin ligases potentially interacting with ALYREF, including NEDD4, RBX1, and RFWD3(Figure S5e, Supporting Information). Co‐immunoprecipitation confirmed ALYREF association with all three. However, DOX treatment selectively increased the interaction between ALYREF and RBX1, without significantly affecting interactions with NEDD4 or RFWD3 (Figure S5f–h, Supporting Information). These findings indicate that DOX promotes RBX1‐mediated ubiquitination and proteasomal degradation of ALYREF.

### 2.6 Doxorubicin Directly Binds to ALYREF Protein

**Figure 6:**
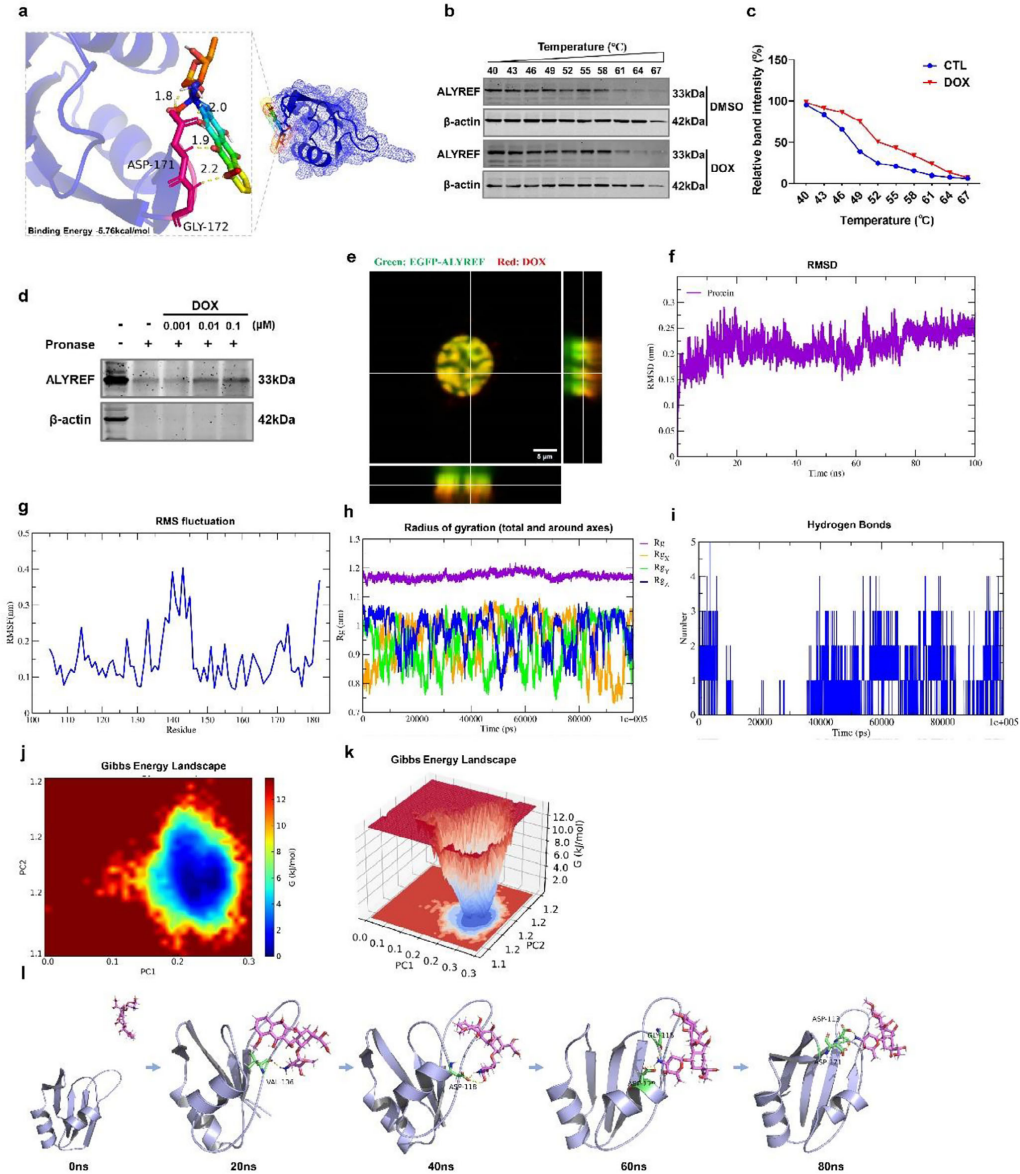
DOX binds directly to ALYREF proteins. a) Molecular docking analysis of ALYREF binding to DOX. b,c) Representative images and quantification curve of CESTA of ALYREF protein levels in CMs treated with DOX or DMSO (*n* = 5). d) DARTS analysis of DOX binding to ALYREF (*n* = 4). e) Live‐cell workstation analysis of EGFP‐ALYREF colocalized with DOX in CMs. Molecular dynamics simulation analysis. f) Root mean square deviation (RMSD) of the protein backbone. g) The root mean square fluctuation (RMSF). h) The radius of gyration (Rg). i) Hydrogen bonding. j,k) Gibbs energy landscape. l) Dynamic ALYREF‐DOX combined simulation diagrams.

DIC has traditionally been attributed to mitochondrial damage in CMs, mainly due to DOX's high affinity for cardiolipin.^[^ ^30^ ^]^ However, DOX can also translocate into the nucleus, where it forms ternary complexes with DNA and TOP2β, leading to DNA double‐strand breaks.^[^ ^9^, ^31^, ^32^ ^]^ In agreement with this mechanism, we assessed the relative nuclear and mitochondrial accumulation of DOX in CMs using surface‐enhanced Raman spectroscopy (SERS), focusing on the compound's characteristic Raman shifts (560 and 2260 cm^−1^). Following DOX treatment, nuclear and mitochondrial fractions were isolated from CMs. Quantitative comparison of DOX peak intensities revealed that mitochondrial DOX levels were approximately 1.5‐fold higher than the DOX standard, whereas nuclear DOX levels were elevated by nearly 6‐fold (Figure S6a–d, Supporting Information). These findings suggest that a substantial amount of DOX enters the nucleus, where it may compromise nuclear function and contribute to cardiotoxicity. Given emerging evidence that small molecules can modulate protein function through direct binding,^[^ ^33^ ^]^ we hypothesized that DOX may interact with ALYREF. Molecular docking simulations predicted that DOX forms hydrogen bonds with residues located in the active pocket region of ALYREF (**Figure** **6**a). To experimentally validate this interaction, we employed cellular thermal shift assay (CESTA) and drug affinity responsive target stability (DARTS) assays. CESTA results demonstrated that DOX treatment significantly increased the thermal stability of ALYREF compared with the DMSO control (Figure 6b,c). Similarly, DARTS analysis revealed enhanced resistance of ALYREF to streptavidin‐mediated degradation in DOX‐treated cells, indicating a protective conformational change upon binding (Figure 6d). Furthermore, fluorescence colocalization studies showed that EGFP‐tagged ALYREF colocalized with DOX in the nuclei of CMs, supporting a physical interaction (Figure 6e). To further characterize this interaction, we performed molecular dynamics simulations. The root means square deviation of the ALYREF backbone, root mean square fluctuation, and radius of gyration remained stable throughout the simulation period, suggesting conformational stability upon binding (Figure 6f–h). Hydrogen bond analysis revealed multiple stable interactions between DOX and ALYREF (Figure 6i), and Gibbs free energy landscape modeling identified two energetically favorable conformations of the ALYREF‐DOX complex (Figure 6j,k). The final docking configuration indicated stable hydrogen bonding between DOX and the aspartate residues at positions 113 and 171 of ALYREF (Figure 6l). Together, these results from molecular docking, thermodynamic assays, and dynamic simulations support a high‐affinity, stable interaction between DOX and ALYREF in CMs.

### 2.7 The D171 Residue of ALYREF Is Essential for Maintaining Cardiomyocyte Genomic Stability

**Figure 7:**
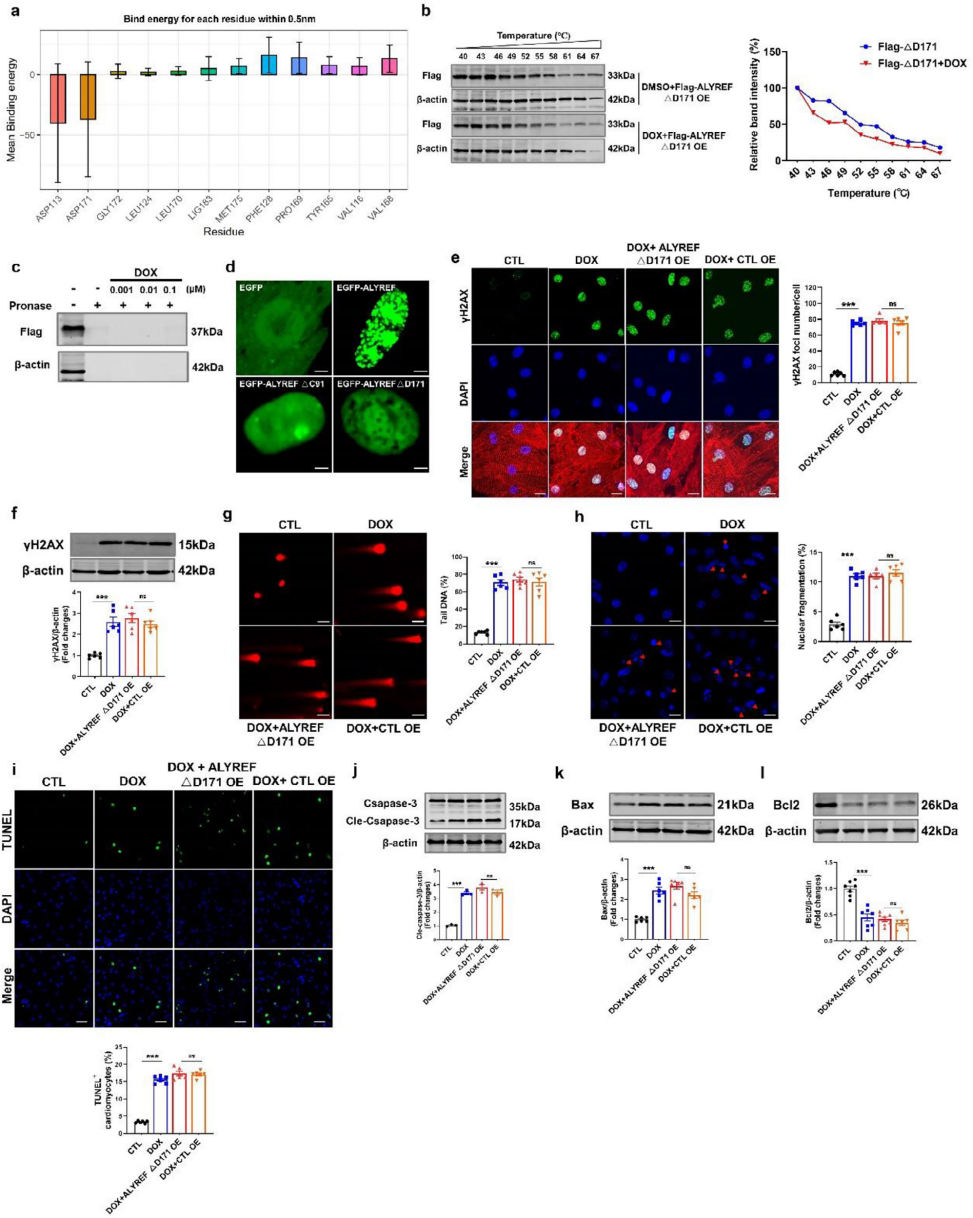
Binding of doxorubicin to ALYREF D171^st^ amino acid induces impaired phase separation and causes DNA damage and apoptosis in cardiomyocytes. Molecular dynamics simulation analysis residue energy. b) Representative images and quantification curve of CESTA of ALYREF △D171 in CMs treated with DOX (*n* = 5). CMs were transfected by Flag‐tagged mutant plasmids with D171 deletion in ALYREF (ALYREF △D171). c) DARTS analysis of DOX binding to ALYREF△D171 (*n* = 4). d) Representative images of the CMs overexpressing EGFP, EGFP‐ALYREF, EGFP‐ALYREF deletion C‐terminal IDRs (EGFP‐ALYREF△IDR), or EGFP‐ALYREF △D171. Scale bars = 10 µm. e–l) CMs transfected with ALYREF△D171 plasmid and then treated with DOX. (e) Representative images and quantification of immunostaining of γH2AX (green), α‐actinin (red, a cardiac marker), and DAPI (blue) in CMs (*n* = 6). Scale bar = 50 µm. (f) Western blot analysis of γH2AX protein levels in CMs (*n* = 6). (g) Representative images and quantification of comet assay in CMs (*n* = 6). Scale bar = 50 µm. (h) Representative images and quantification of immunostaining of the DAPI in CMs (*n* = 6). Scale bar = 50 µm. (i) Representative images and quantification of TUNEL staining in CMs (*n* = 6). Scale bar = 50 µm. (j–l) Western blot analysis of Cleaved‐Caspase‐3 (*n* = 3), Bax (*n* = 6), and Bcl2 (*n* = 7) protein levels in CMs. Data are presented as mean ± SEM. (e–l) Statistical analysis by one‐way ANOVA with Tukey's multiple comparisons test. ^***^ *p* < 0.001.

Molecular docking analysis revealed that DOX stably interacts with ALYREF at residues D171 and D113 (Figure 6l). Residue energy profiling further confirmed that D171 and D113 exhibit the highest binding affinity for DOX, with D171 showing the strongest interaction (**Figure** **7**a). To functionally dissect these interactions, we generated Flag‐tagged ALYREF deletion mutants lacking either D171 or D113 (ALYREF ΔD171 or ΔD113) and employed DARTS and CESTA to assess binding. Deletion of D171 abolished the interaction between ALYREF and DOX, whereas DOX remained capable of binding to the ΔD113 mutant (Figure 7b,c; Figure S7a, Supporting Information), indicating that D171 is the primary site for DOX binding.

Notably, residue D171 is located within the C‐terminal IDRs of ALYREF (Figure 5d). Deletion of the C‐terminal IDRs disrupted the condensate‐forming capacity of ALYREF, suggesting that D171 is critical for LLPS (Figure 7d). Importantly, deletion of D171 alone recapitulated this phase separation defect, further implicating this residue in the structural integrity of ALYREF condensates (Figure 7d). To determine whether the loss of D171 affects ALYREF's genomic protective function, we overexpressed ALYREF ΔD171 in CMs subjected to DOX treatment. Unlike wild‐type ALYREF, the ΔD171 mutant failed to rescue DOX‐induced increases in γH2AX expression, comet tail DNA percentage, and nuclear fragmentation (Figure 7e–h). Additionally, ALYREF ΔD171 did not prevent DOX‐induced apoptosis in CMs, as shown by TUNEL staining, flow cytometry, and altered expression of apoptosis‐related proteins (Figure 7i–l; Figure S7b,c, Supporting Information). In summary, D171 is a dual‐function residue that serves as the critical binding site for DOX and is indispensable for the phase separation properties of ALYREF. Its deletion disrupts ALYREF condensate formation and compromises its ability to preserve genomic stability in cardiomyocytes.

### 2.8 Effect of ALYREF on Tumor Proliferation is Independent of its Phase Separation Ability

ALYREF has been reported to promote tumor progression via m⁵C‐mediated RNA regulation.^[^ ^27^, ^34^, ^35^ ^]^ However, our findings indicate that overexpression of the mutant ALYREF 163 site plasmid is still resistant to DOX‐induced DNA damage and apoptosis in CMs that mutation of its K163 does not affect its ability to form phase‐separated condensates (Figure 4; Figure S8a, Supporting Information). Notably, clustered condensates formed by full‐length human ALYREF (EGFP‐ALYREF(H)) and the m⁵C‐binding‐deficient mutant (EGFP‐ALYREF m⁵C mutant (H)) were also disrupted by DOX and 1,6‐HEX treatment, indicating that condensate formation is not dependent on m⁵C binding (Figure S8b, Supporting Information). Subsequently, we evaluated the effects of ALYREF on tumor growth and cardiac function in a DIC model. We utilized the A549 lung cancer model, in which ALYREF has been shown to facilitate tumor proliferation through m⁵C‐dependent mechanisms.^[^ ^14^ ^]^ A DIC model was established in A549 tumor‐bearing mice (Figure S8c, Supporting Information). Tumor volume and weight did not differ significantly between mice treated with AAV9‐ALYREF m⁵C mutant and those treated with AAV9‐EGFP, regardless of DOX exposure (Figure S8d–f, Supporting Information). Notably, the ALYREF K163A mutant abolished its tumor‐promoting effect, yet preserved its cardioprotective function. The mice expressing the ALYREF K163A mutant exhibited improved myocardial contractility, as reflected by enhanced EF and FS compared with controls (Figure S8g–i, Supporting Information). In conclusion, the above results indicate that ALYREF functions as an m^5^C reader to promote tumor growth independent of its LLPS ability. The ALYREF K163A mutan still retains its phase‐separated condensate state and can protect myocardial function in mice.

### 2.9 Doxorubicin Compromises Genome Stability by Inducing Dissociation of the NARC1 Complex

Next, we would like to further elucidate the molecular mechanisms underlying the disruption of ALYREF phase separation and DNA damage. Our data demonstrate that DOX readily enters the nuclei of CMs, where it directly binds to ALYREF and disrupts its phase separation capacity without impairing nuclear localization. This nuclear‐restricted perturbation implicates DNA damage‐associated pathways in DIC . Given that ALYREF exerts cardioprotective effects predominantly through safeguarding genome integrity, its downstream effectors are likely to participate in DNA damage response (DDR) and genome maintenance pathways. Interestingly, a previous study by Munschauer et al. identified ALYREF as a core component of the NARC1 complex in HCT116 cells, where it contributes to genomic stability.^[^ ^15^ ^]^ The known constituents of NARC1 (ALYREF, RBMX, CDC5L, TOP1, and PRPF19) are all nuclear‐localized, involved in DDR, and conserved across diverse cell types.^[^ ^36^, ^37^, ^38^, ^39^ ^]^

**Figure 8:**
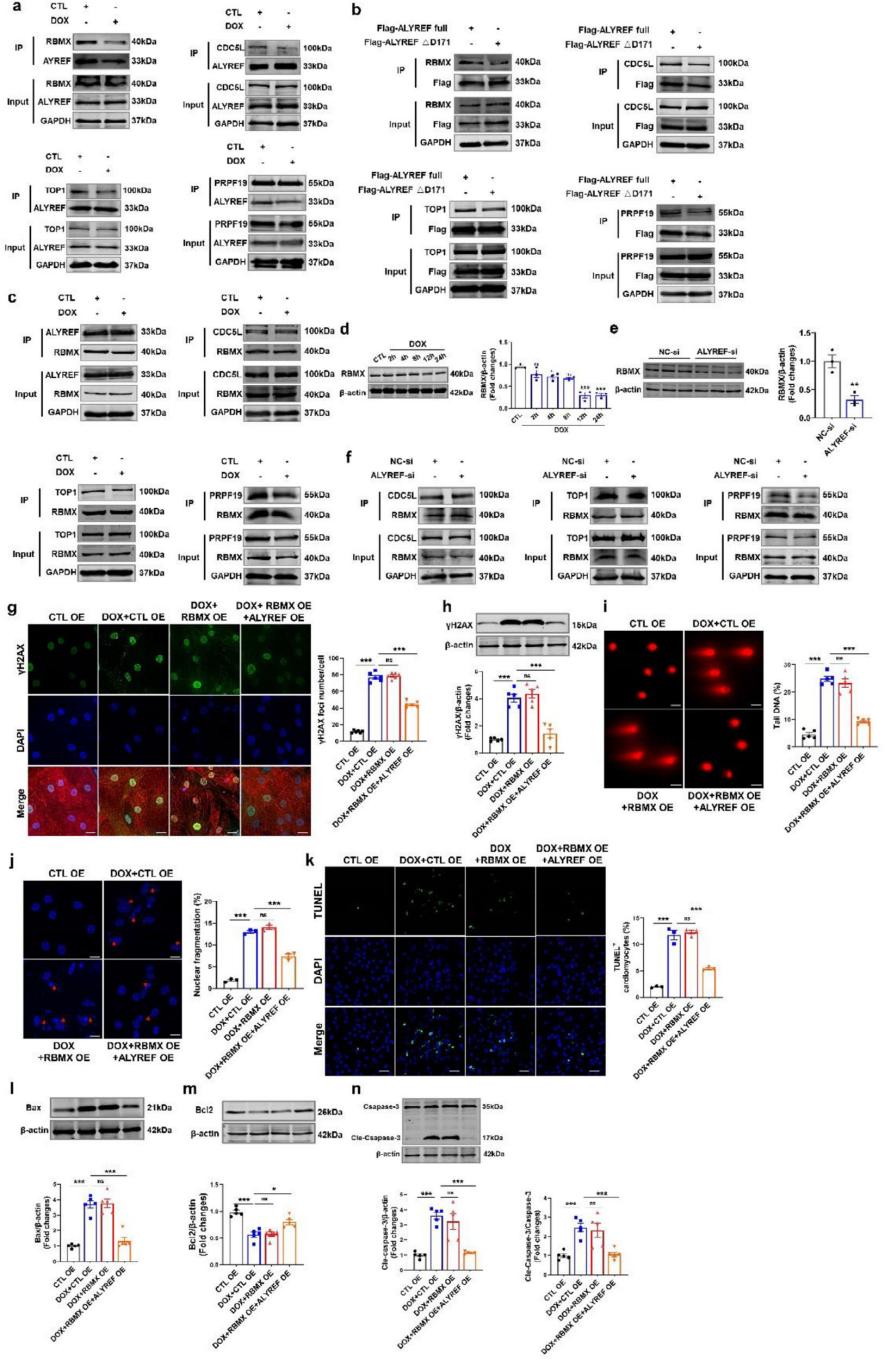
Doxorubicin affects genome stability by causing dissociation of the NARC1 complex. a,b) Immunoprecipitation analysis of ALYREF interaction with RBMX, CDCL5, TOP1, and PRPF19 in CMs treated with DOX or overexpressing Flag‐ALYREF D171 plasmid. c) Immunoprecipitation analysis of RBMX interaction with ALYREF, CDCL5, TOP1, and PRPF19 in CMs treated with DOX. d) Western blot analysis of RBMX protein levels in CMs treated with DOX at different time points (*n* = 3). e) Western blot analysis of RBMX protein levels in CMs after transfected with ALYREF siRNA (*n* = 3). f) Immunoprecipitation analysis of RBMX interaction with CDCL5, TOP1, PRPF19 in CMs after transfected with ALYREF siRNA. g–n) CMs after cotransfection with RBMX and ALYREF plasmid and then treated with DOX. (g) Representative images and quantification of immunostaining of γH2AX (green), α‐actinin (red, a cardiac marker), and DAPI (blue) in CMs (*n* = 6). Scale bar = 50 µm. (h) Western blot analysis of γH2AX protein levels in CMs (*n* = 6). (i) Representative images and quantification of comet assay in CMs (*n* = 5). Scale bar = 50 µm. (j) Representative images and quantification of immunostaining of the DAPI in CMs (*n* = 3). Scale bar = 50 µm. (k) Representative images and quantification of TUNEL staining in CMs (*n* = 3). Scale bar = 50 µm. (l–n) Western blot analysis of Bax (*n* = 5), Bcl2 (*n* = 5), and Cle‐Caspase‐3 (*n* = 5) protein levels in CMs. Data are presented as mean ± SEM. Statistical analysis was performed by (d,e) Student's *t*‐test, and (g–n) by one‐way ANOVA with Tukey's multiple comparisons test. ^*^ *p* < 0.05, ^**^ *p* < 0.01, and ^***^ *p* < 0.001.

Consistent with this, co‐immunoprecipitation confirmed the presence of the NARC1 complex in CMs (**Figure** **8**a). DOX treatment of CMs for 2 h induced impaired phase separation of ALYREF, which resulted in decreased binding of ALYREF to RBMX, TOP1, and CDC5L, but not to PRPF19 (Figure 8a). Similarly, mutation of the D171 residue in ALYREF, the identified DOX binding site, also disrupted NARC1 complex assembly (Figure 8b). As RBMX has been reported to be critical for maintaining NARC1 complex stability,^[^ ^15^ ^]^ we examined its protein–protein interactions following DOX‐induced disruption of ALYREF condensates. Notably, impaired ALYREF phase separation diminished RBMX binding to PRPF19 and ALYREF, while its interactions with TOP1 and CDC5L remained intact (Figure 8c). Furthermore, both DOX exposure and ALYREF depletion led to downregulation of RBMX expression in CMs (Figure 8d,e). ALYREF knockdown abolished RBMX binding to all other NARC1 components, resulting in complete complex dissociation (Figure 8f). These findings indicate that DOX‐induced disruption of ALYREF phase separation destabilizes ALYREF‐RBMX and RBMX–PRPF19 interactions, thereby initiating disassembly of the NARC1 complex. To further assess the functional interplay between ALYREF, RBMX, and DOX‐induced NARC1 dissociation, we evaluated whether RBMX overexpression could compensate for ALYREF loss in protecting against DOX‐induced DNA damage. However, overexpression of RBMX alone did not reduce DNA damage or apoptosis in DOX‐treated CMs. In contrast, coexpression of ALYREF and RBMX significantly alleviated DOX‐induced DNA damage and apoptosis (Figure 8g–n; Figure S9a, Supporting Information). Similarly, in a mouse model of DIC, RBMX overexpression alone failed to confer cardio protection, whereas coexpression of RBMX and ALYREF markedly improved cardiac function and reduced myocardial injury (Figure S9b–r, Supporting Information). Together, these findings suggest that DOX‐induced binding to ALYREF disrupts its condensate state, leading to progressive disassembly of the NARC1 complex. ALYREF degradation in turn reduces RBMX expression, which further destabilizes NARC1, ultimately promoting genome instability. These data establish ALYREF as a critical scaffold for maintaining NARC1 complex integrity during DOX‐induced cardiotoxic stress.

## 3 Discussion

Although DIC has been extensively linked to myocardial oxidative stress and mitochondrial dysfunction, the nuclear mechanisms by which DOX mediates cardiomyocyte injury remain poorly defined. In this study, we identified a previously unrecognized mechanism in which DOX directly binds to D171 of ALYREF, disrupting its LLPS and promoting its ubiquitin‐mediated degradation. We demonstrated that the LLPS capacity of ALYREF is essential for maintaining the integrity of NARC1 during DIC. DOX binding to ALYREF impairs its condensate‐forming ability, resulting in the progressive dissociation of the NARC1 complex and ultimately triggering DNA damage and apoptosis in CMs. IDRs are known to drive LLPS through multivalent, weak interactions facilitated by their flexible conformational architecture.^[^ ^40^ ^]^ Sequence analysis revealed that ALYREF contains two IDRs, supporting its strong intrinsic propensity for phase separation. Consistently, we observed that ALYREF formed dynamic, droplet‐like condensates exhibiting spherical morphology, fusion behavior, and reversibility upon contact. Deletion of the C‐terminal IDRs abolished condensate formation, confirming that this region is essential for ALYREF‐mediated phase transitions.

Our study focused on the pathological effects of DOX on ALYREF phase separation. We demonstrated that DOX selectively binds to D171 within the C‐terminal IDRs of ALYREF, perturbing its condensate structure. However, the role of the N‐terminal IDRs in regulating ALYREF phase separation remains unexplored. Future studies are warranted to dissect how the N‐terminal IDRs contribute to the biophysical and functional properties of ALYREF condensates, which may refine our understanding of its broader biological roles. To further investigate the nature of ALYREF condensates, we utilized 1,6‐HEX, a small molecule known to disrupt LLPS driven by hydrophobic, but not electrostatic, interactions.^[^ ^29^ ^]^ Exposure to 1,6‐HEX effectively abolished ALYREF condensates, indicating that ALYREF undergoes phase separation primarily via hydrophobic interactions. Moreover, phase‐separated condensate formation is concentration‐dependent, requiring a critical threshold for self‐assembly. During progressive DOX‐induced injury, ALYREF protein levels in CMs declined, reducing local concentrations below the phase‐separation threshold and forming a feed‐forward loop that further antagonized condensate formation and exacerbated cellular damage. Previous work by Guttman et al. demonstrated that ALYREF is a key component of the NARC1 complex, which is required for genome stability. The RNA‐binding protein RBMX, another component of NARC1, mediates LncNORAD interactions with partner proteins and is indispensable for NARC1 complex integrity.^[^ ^15^ ^]^ Notably, genomic stability cannot be maintained when RBMX is absent or when LncNORAD lacks RBMX‐binding capacity. In our study, we showed that ALYREF LLPS is required to stabilize the NARC1 complex in the context of DIC. DOX directly targets ALYREF, promoting condensate dispersion and partial disassembly of the complex. Importantly, overexpression of RBMX conferred resistance to DOX‐induced DNA damage only when ALYREF was present, underscoring that ALYREF is the direct target of DOX and that its condensate state is critical for preserving NARC1 complex integrity under cardiotoxic stress.

In a study by Ding et al., ALYREF was identified as an m⁵C reader that cooperates with NSUN2 to enhance m⁵C methylation of Nrf2 mRNA, thereby suppressing antioxidant responses and contributing to DOX‐induced hepatocyte injury.^[^ ^41^ ^]^ In contrast, our results suggest that ALYREF is primarily active through its phase‐separating ability to resist DIC, overexpression of the ALYREF K163 mutant still resists DOX‐induced DNA damage and apoptosis in CMs. Previous reports have shown that DOX enters the nucleus of CMs and forms ternary complexes with TOPIIβ and DNA.^[^ ^42^ ^]^ Consistent with this, we observed greater nuclear accumulation of DOX in CMs than in mitochondria under DIC conditions (Figure S6a–d, Supporting Information). Despite this, most mechanistic studies to date have focused on mitochondrial effects of DOX in CMs, while the nuclear targets and mechanisms mediating myocardial injury remain poorly defined. Our study supports a nuclear‐centric mechanism in which DOX directly binds to ALYREF, leading to disruption of its phase‐separating condensates and promoting its ubiquitination and degradation. This disturbance in ALYREF phase separation compromises genome stability and induces DNA damage and apoptosis, which we propose to be a primary mechanism underlying ALYREF‐mediated protection in DIC. Although ALYREF is the only known nuclear m⁵C reader involved in mRNA maturation and export, it may also play a secondary role in modulating DOX‐induced reactive oxygen species generation and mitochondrial dysfunction. This could occur through transcriptional regulation of antioxidant enzymes or mitochondrial factors, linking ALYREF's m⁵C‐related functions to broader cellular responses. While this hypothesis was not explored in this study, it represents a valuable direction for future investigation aimed at fully delineating ALYREF's multifaceted role in DIC.

In conclusion, our findings demonstrate that nuclear DOX directly binds to ALYREF, inducing condensate disruption and subsequent ubiquitination and degradation. ALYREF maintains genomic stability primarily through its LLPS capacity, and impairment of this phase behavior results in DNA damage and cardiomyocyte apoptosis. These results reveal a previously underappreciated mechanism by which DOX impairs nuclear integrity via ALYREF and offer a novel framework for understanding the molecular pathogenesis of DIC.

## 4 Experimental Section

### Animals

Male C57BL/6J mice were purchased from Changsheng Bio‐technology (Changchun, China). Female nude mice were purchased from Qiguan Biotechnology (Harbin, China). Cardiac‐specific ALYREF knockout (ALYREF CKO) mice on C57BL/6 background constructed with CRISPR/Cas9 were purchased from Cyagen (Suzhou, China). All mice were housed in temperature‐controlled facilities at 22 ± 2 °C in a controlled 12 h light‐dark cycle and fed standard mouse chow and tap water. For experiments with knockout mice, control mice of the same age/sex from littermates or sibling mating were used. Animal experimental protocols were approved by the Ethical Committee of Harbin Medical University for Animal Research (Approval No: SYDW2023‐107).

### Generation of Cardiac‐Specific ALYREF Knockout Mice

The CRISPR/Cas9 system was used to generate cardiac‐specific ALYREF knockout mice were generated by Cyagen (Suzhou, China). Cardiac‐specific conditional ALYREF deficiency mice (ALYREF^flox/flox^/αMHC‐Cre) were generated by crossing ALYREF^flox/flox^ mice with αMHC‐Cre mice. And the adult mice (8‐week‐old) received continuous intraperitoneal injections of tamoxifen (30 mg kg^−1^ day^−1^) for 5 days to induce ALYREF knockout in cardiac. Mice will be used for subsequent experimental analysis after2 weeks.

### Construction ALYREF Cardiac‐Specific Overexpression Mice by Adeno‐Associated Virus 9

8 weeks male C57BL/6 mice received tail vein injections of 2 × 10^12^ vg of ALYREF‐overexpressing adeno‐associated virus‐9 genome particles (AAV9‐EGFP‐cTnT‐ALYREF) containing the cTnT promoter and EGFP (Hanbio Biotechnology, Shanghai, China).The control group received injections of AAV9‐EGFP. Mice will be used for subsequent experimental analysis after 4 weeks.

### Doxorubicin‐Induced Cardiotoxicity Model Mice

To induce the DIC model, male or female C57BL/6J mice were injected intraperitoneally with DOX (5 mg kg^−1^) (MB1087, Meilunio, China) once a week for 4 weeks. Although mostly administered intravenously in clinical practice, DOX‐induced cardiomyopathy mouse models are widely used for intravenous administration because of the advantages of stable serum drug concentration, high success rate, and ease of reproducing DOX‐induced cardiomyopathy in mice.^[^ ^43^ ^]^ Echocardiography was used to analyze structural and functional changes in the heart before and after administration of DOX treatment, respectively. The investigators followed standard laboratory procedures for randomization and analyzed data in a blinded manner. The investigators were blinded to the genotype of individual animals during the experiments and evaluation of the results.

### Echocardiography

Cardiac function was assessed using a Vevo 2100 high‐resolution small animal ultrasound imaging system and a MicroScan MS 250‐0206 probeMice were anesthetized using inhalation of 1% isoflurane and placed on the heated platform. M‐mode echocardiographic short‐axis was used to assess left ventricular ejection fraction, left ventricular fractional shortening, left ventricular end diastolic diameter (LVIDd), and left ventricular end systolic dimension (LVIDs).

### Cell Culture: Neonatal Mouse Cardiomyocytes, Fibroblasts, and Endothelial Cells

The experimental method was carried out mainly with reference to this protocol.^[^ ^44^ ^]^ Briefly, the hearts of newborn mice were cut into chunks and then digested with trypsin (T1300, Solarbio, China). The cell suspension was collected by centrifugation and incubated at 37 °C, 5% CO_2_ for 90 min. Finally, the cell ratio was adjusted by adding in Dulbecco's modified Eagle's medium (DMEM; Life Technologies, USA) supplemented with 10% fetal bovine serum (FBS; Gibco, USA) and 1× penicillin and streptomycin (Life Technologies, USA) and the plates were spread for culture.

### Cell Line Culture

HEK293T and A549 cells were purchased from Anwei‐sci Cell Center (Shanghai, China) and cultured in DMEM (Life Technologies, USA) supplemented with 10% FBS (Gibco, USA) and 1× penicillin and streptomycin (Life Technologies, USA).

### Human‐Induced Pluripotent Stem Cell‐Derived Cardiomyocytes

The hiPSC‐CMs were obtained from the National Stem Cell Resource Center, and were maintained in an E8 medium (CA4024106, CELLAPY) at 37 °C and 5% CO_2_. When cells reached 80% confluence, they were incubated in basal medium containing RPMI 1640 (C11875500BT, Thermo Fisher Scientific, MA, USA) plus B27 supplement minus insulin (A1895601; Thermo Fisher, MA, USA). On the 1 day of the differentiation, 6 µmol L^−1^ CHIR‐99021 (HY‐10182; MCE, Monmouth Junction, NJ, USA) was added to the medium. On the 3 days of differentiation, 2 µmol L^−1^ Wnt‐C59 (HY‐15659; MCE, Monmouth Junction, NJ, USA) was added to the medium. On day 7 of differentiation, the medium was changed to RPMI 1640 and B27 supplement (17504044; Thermo Fisher, USA). Beating CMs were observed after day 7 of differentiation, and as differentiation progressed to days 8–9, the number of beating CMs increased and could be processed for subsequent experiments.

### Synthesis and Transfection of Overexpression Plasmids and siRNAs

The siRNAs of ALYREF were designed and synthesized by SevenBiotech (Beijing, China). Mouse‐derived ALYREF, ALYREF △C91, ALYREF m^5^C mutant, ALYREF △D171 and △D113 plasmids and human‐derived ALYREF, ALYREF m^5^C mutant were constructed by HonorGene (Changsha, China). For fluorescently labeled gene expression in CMs, plasmids with ALYREF or one of its variants were constructed by subcloning the corresponding vector DNAs into the pEGFP. Transfections were performed by using Lipofectamine RNAiMAX, Lipofectamine 2000 (Invitrogen, USA). Transfections have been performed according to the manufacturer's protocols.

**Table:** 

ALYREF siRNA	5′‐GGAACUCUUUGCUGAAUUUTT‐3′
	5′‐ AAAUUCAGCAAAGAGUUCCTT‐3′
NC‐siRNA	5′‐UAUCACAACAGAUCCACUGTT‐3′
	5′‐CUAAGGAACAACAGAGCAATT‐3′

### Drug Treatment: Induction of DIC Cell Model

When the cell growth status was suitable, the culture medium was discarded, PBS buffer was washed once, and DOX (1 µmol L^−1^) (MB1087, Meilunbio, China) solution was configured with DMEM and added into the cell well plate. Finally, the well plates were put into a 37 °C, 5% CO_2_ cell culture incubator for culture. According to the experimental design, cells were collected at different time points of DOX induction for subsequent experiments.

### Drug Treatment: Cycloheximide (CHX)

When the cell growth status was suitable, the culture medium was discarded, PBS buffer was washed once, 1 µmol L^−1^ DOX solution configured with DMEM was added to the cell well plates, CMs were pretreated with DOX and cultured in a 37 °C, 5% CO_2_ cell incubator for 3 h. Subsequently, the solution in the well plates was discarded, PBS buffer was rinsed once, and 5 µmol L^−1^ CHX solution configured with DMEM was added to the well plates, which were cultured in the cell CMs were collected at 0, 4, and 8 h after the addition of CHX (HY‐12320, MedChemExpress, USA), and the proteins were extracted and subjected to Western blot assay to detect the changes of ALYREF protein expression in each group of cells.

### Drug Treatment: Proteasome Inhibitor (MG‐132)

When the cell growth status was suitable, the culture medium was discarded, washed once with PBS buffer, 20 µmol L^−1^ proteasome inhibitor MG‐132 solution (HY‐13259, MedChemExpress, USA) was configured with DMEM, CMs were pretreated with MG‐132, and cultured in a cell culture incubator at 37 °C with 5% CO_2_ for 12 h. Subsequently, the solution in the well plate was discarded, rinsed once with PBS buffer, and 1 µmol L^−1^ DOX solution was configured with DMEM and added to the cell well plate, and cultured in the cell culture incubator. The cells were then incubated in a cell culture incubator for 12 h. Subsequently, the extracted proteins were collected from each group of cells and subjected to Western blot assay to detect the changes of ALYREF protein expression in each group of cells.

### Drug Treatment: Chloroquine (CQ)

When the cell growth status was suitable, the culture medium was discarded, the PBS buffer was washed once, and 20 µmol L^−1^ CQ concentrated storage solution (HY‐17589A, MedChemExpress, USA) was configured with DMEM and added to the well plates, the CMs were pretreated with CQ, and put into the cell incubator at 37 °C, 5% CO_2_ for 12 h. Subsequently, the solution in the well plates was discarded, and the cell plates were rinsed with PBS buffer, and the 1 µmol L^−1^ DOX solution was configured with DMEM and added to the cell well plates and incubated for 12 h in a cell culture incubator. After incubator for 12 h, the process was followed by collecting the extracted proteins from each group of cells for Western blot assay to detect the changes of ALYREF protein expression in each group of cells.

### Comet Assay

1% agarose gel preheated at 100 °C was dropped onto the slide, and it was allowed to stand at room temperature for 10 min; the cell suspension was mixed with 0.6% low melting point agarose preheated at 40 °C, the mixture was dropped onto the first layer of the gel plate, and allowed to stand at room temperature for 10 min; 0.6% low melting point agarose gel preheated at 40 °C was dropped onto the top layer of the gel, and permitted to stand at room temperature to solidify completely; The slide was lysed into cell lysis buffer (2.5 m NaCl, 100 mm disodium EDTA, 10 mm tris‐base, pH 10, 10% DMSO, and 1% Triton X‐100) at 4 °C for 1 h; it was rinsed with PBS buffer for three times, then the slide was put into alkaline electrophoresis buffer (300 mm NaOH, 1 mm disodium EDTA) and it was deconvoluted at room temperature for 20 min, so that the DNA double strand can be dehelical zed to form a single strand, which is easy to be migrated in the electrophoresis field 0.5× TAE was precooled at 4 °C, and then poured into the electrophoresis tank. It was put in gel slides. The power parameter was set at 50 V for 10 min, and electrophoresis was carried out under low temperature conditions. After electrophoresis, the slides were washed three times with PBS buffer. Then PI staining solution was added dropwise on the slide, covered with a coverslip, and incubated at room temperature and protected from light for 20 min, and the cellular DNA breakage was observed at the excitation light wavelength of 594 nm using a fluorescence microscope (IX73P1F, OLYMPUS, Japan). Tail moment of CMs was counted by CASP software.

### Preparation of Enhanced Substrate and SERS Detection

Silver nanoparticles were prepared by reducing silver nitrate with sodium citrate. Specifically, 0.07 g of silver nitrate was dissolved in 400 mL of deionized water. The resulting solution was heated to 98 °C, followed by the addition of 12 mL of a 0.01 g mL^−1^ sodium citrate solution. The reaction was allowed to proceed for 5 min before being stopped. The mixture was then centrifuged at 6500 rpm for 10 min. After discarding the supernatant, the obtained nanoparticles were resuspended and mixed in a 1:1 ratio with a 10 mmol L^−1^ KI solution for further use.

The samples were mixed with methanol in a 1:6 ratio, then vortexed for 1 min and allowed to stand for 5 min. The mixture was centrifuged at 12000 rpm for 10 min, and the supernatant was carefully collected. The samples were then dried under a stream of nitrogen gas. Afterward, 200 µL of deionized water was added to reconstitute the samples, followed by vertexing for 1 min. The samples were centrifuged again at 12 000 rpm for 10 min, and the supernatant was collected for analysis. The characteristic peaks of DOX were observed at 450, 1206, and 1436 cm^−1^.^[^ ^45^ ^]^

### Western Blot

The total protein was extracted from CMs with RIPA lysis buffer (P0013B, Beyotime, China), and a BCA assay (P0010, Beyotime Biotechnology, China) was performed to quantify the protein concentrations. The equal amount of protein samples was loaded and resolved on 12.5% SDS‐PAGE gel, and transferred to nitrocellulose membrane (Millipore, USA). The membranes were blocked in 5% milk and then reacted overnight with primary antibodies against ALYREF (1:500, AB202894, Abcam, UK), RBMX (1:200, AB190352, Abcam, UK), γH2AX (1:1000, AB81299, Abcam, UK), PRPF19 (1:500, Thermo Fisher, USA), Caspase‐3 (1:750, 9662s, Cell Signaling Technology, USA), Bax (1:750, CY5059, Abways, USA), Bcl2 (1:750, CY6717, Abways, USA), Flag (1:1000, YM3808, Proteintech, China), ACTB (1:1000, TA‐09, ABclonal, USA), GAPDH (1:1000, AB0037, Abways, USA) at 4 °C. The membranes were finally incubated with secondary antibodies for 1 h at RT and scanned with Odyssey (LI‐COR Biosciences, USA).

### Co‐Immunoprecipitation (Co‐IP)

After CMs were induced by 1 µmol L^−1^ DOX for 2 h, cell precipitates were collected and added to RIPA lysate (P0013B, Beyotime, China), and the protein concentration of the supernatant was detected by BCA method. The protein concentration was calculated according to the standard curve formula, and an equal mass of protein solution was taken from each group, added with ALYREF antibody (10 µg, AB202894, Abcam, UK) or control IgG (cat. no‐3900s, Cell Signaling) and incubated overnight at 4 °C on a rotary shaker. The “antigen–antibody” mixture was added to the protein A/G magnetic beads washed three times with Co‐IP washing buffer (PBS buffer containing 0.5% Triton X‐100, pH 7.4), and incubated on a vertical shaker for overnight at 4 °C. On the next day, the magnetic bead–protein–antibody complex was removed and washed four times with Co‐IP elution buffer (PBS buffer containing 0.5% Triton X‐100 and 0.15 mol L^−1^ glycine, PH 7.4), and protein denaturation was performed by adding a certain amount of SDS‐PAGE protein loading buffer and heating at 95 °C for 7 min. SDS‐PAGE electrophoresis was performed after boiling the samples for denaturation.

### Cellular Thermal Shift Assay

CMs were collected by trypsin digestion, centrifuged at 300 g for 3 min, and the supernatant was discarded to collect the cell precipitates; then the cell precipitates were resuspended with precooled PBS, and after the second centrifugation, the supernatant was discarded, and ≈440 µL of PBS buffer was added to each tube and blown well; subsequently, the cell suspensions were divided into 200 µL EP tubes, divided into 10 portions of 40 µL each; the cells were heated in correspondence to the temperature gradient from 40 to 67 °C for 3 min, let it stand at room temperature for 3 min, then it was put into liquid nitrogen, and the above operation was repeated for three times. After that, the parameters of cryogenic centrifuge were set at 20 000 × *g*, 4 °C, centrifuged for 20 min, centrifuged the cell samples, 20 µL cell supernatant was transferred to the corresponding labeled new EP tubes, added 5 µL loading, vortex centrifuge; the samples were cooked at 70 °C for 10 min, and then the samples were analyzed by protein electrophoresis.

### Drug Affinity Responsive Target Stability Assay

Trypsin digestion was performed to collect CMs, and appropriate amount of protein lysate was added and placed on ice to fully lyse for 10 min. Low‐temperature centrifuge at 18 000 × *g*, centrifugation was performed for 10 min, followed by transferring the cellular supernatant to a new EP tube. According to the volume of the supernatant in the tube, 10× TNC was added in the ratio of 1:10, mixed well, and the protein concentration was detected by BCA assay; the protein whose concentration had been detected, 29.7 µL of supernatant was aspirated, and 0.3 µL DOX was added to the DOX group, except the Input group; 0.3 µL DMSO solution was added to the CTL group, and it was upside down at room temperature for 1 h. After the incubation was completed, the samples were divided into 20 µL groups, and 2 µL of TNC diluted streptavidin solution (solubilized streptavidin:protein = 1:1000) was added to each tube; the Input group was added with an equal volume of TNC solution; the samples were left to stand at room temperature for 15 min; 2 µL of 20× protease inhibitor was added to each group, and it was left to stand on ice for 1 min; 6 µL of 5× SDS‐PAGE Loading Buffer was added to each group, and the samples were boiled at 70 °C for 10 min. The samples were boiled at 70 °C for 10 min and detected by protein electrophoresis.

### Live‐Cell Imaging of Neonatal Cardiomyocytes

The CMs was transfected with EGFP‐ALYREF, EGFP‐ALYREF△D171 EGFP‐or ALYREF△C91 plasmid and scanned under a confocal laser scanning microscope (FV10i, Olympus, Japan). The time‐lapse images of EGFP‐ALYREF were taken continuously at a time interval of 240 s with the same environment and parameters. In this experiment, CMs transfected with EGFP‐ALYREF plasmid were induced with 5% 1,6‐hexanediol for 10 min or 1 µmol L^−1^ DOX for 2 h. ImageJ software (NIH) was used to analyzing the fluorescence intensity of EGFP‐ALYREF in CMs.

### Fluorescence Bleaching Recovery Assay (FRAP)

FRAP analysis was performed using the FRAP module of a confocal laser scanning microscope (Zeiss LSM 800, German). A 488 nm laser was used to bleach EGFP‐ALYREF. The bleaching was focused on the region of interest using 100% laser power, and time‐lapse images were acquired and fluorescence intensity was measured. Background intensity was subtracted and values relative to the prebleaching time point were reported. The images were scanned 3 times and about 300 times before and after bleaching until the fluorescence signal was stable. The “fluorescence recovery–time” curves of EGFP‐ALYREF before and after bleaching were fitted using Prism 8.0 software.

### In Vitro pSMARt‐I‐ALYREF Expression, Protein Purification, and Droplet Formation Assay

The pSMARt‐I‐ALYREF plasmid was transformed into Escherichia coli BL21(DE3) cells according to standard supplier protocols. The monoclones on the transformation plate were selected and grown to an optical density (OD600) of 0.6–0.8. The isopropyl‐β‐d‐thiogalactoside (Sigma) was added to the culture to a final concentration of 0.4 mmol L^−1^ and shaken overnight at 15 °C to induce the expression of fusion protein. After ultrasonic fracturing, the fusion proteins were purified by Ni‐IDA‐Sepharose Cl‐6B affinity chromatography. The eluted proteins were dialyzed overnight with 20 mmol L^−1^ Tris‐HCl, 0.15 mol L^−1^ NaCl, pH 8.0 and concentrated with an ultrafiltration Centrifugal Filters (Millipore). The purified protein was concentrated to ≈3.3 mg mL^−1^, 20 mmol L^−1^ Tris, pH 8.0.

For droplet formation assay in vitro, 5 mmol L^−1^ purified mouse‐derived ALYREF protein was mixed with phase separation buffer containing 250 mmol L^−1^ Tris (pH 8.0), 10% PEG‐8000, and incubated at room temperature for 20 min. Finally, 200 µL of the mixture for each sample was transferred to a glass dish to be left to stand for 30 min and imaged using a confocal laser scanning microscope (Zeiss LSM 800, German) phase contrast module. Images were recorded over a period of time and analyzed for ALYREF protein droplet aggregation mobility using Zeiss software.

### ELISA

Mouse plasma samples were analyzed by ELISA, and the experiments were performed according to the instructions of the ELISA kit. The Cardiac troponin Assay Kit (JL20611, Shanghai Jianglai Biotechnology, China) was used to measure the plasma TNT level. The Creatine kinase MB isoenzyme Assay Kit (JL12422, Shanghai Jianglai Biotechnology, China) was used to measure the plasma CK‐MB level. Briefly, the standards and samples were added to the precoated plate and incubated for 2 h at 37 °C; Wash buffer wash repeated five times, then biotin‐conjugated antibody working solution was added and incubated for 1 h at 37 °C; Wash buffer wash repeated three times, then streptavidin–HRP working solution was added and incubated for 30 min at 37 °C; Wash buffer wash repeated three times, then TMB solution was added and incubated for 20 min at 37 °C and protected from light, then termination solution was added and the absorbance at 450 nm was detected using an enzyme meter; the standard curve was plotted and the calculation formula was based on the absorbance measured from the standards.

### Histological Analysis

Heart was fixed by 4% paraformaldehyde (Solarbio, China), then dehydrated by gradient xylene, paraffin‐embedded, and myocardial tissues of 5 µm thickness were cut. H&E (G1120, Solarbio, China) staining was used to evaluate heart morphology and tissue structure. Masson's trichrome staining (G1340, Solarbio, China) and Sirius red staining (G1472, Solarbio, China) were performed to evaluate cardiac collagen deposition. Daylight microscopy (BX53F, OLYMPUS, Japan) was used to take images of heart tissue and representative images were selected based on the average of the heart sizes of each group of mice to accurately represent the average of each group.

### Wheat Germ Agglutinin (WGA) Staining

WGA staining was used to analyze single cell area. Cardiac tissues embedded with OTC compounds were first sectioned to a thickness of 4 µm. The heart tissue sections were fixed with acetone at room temperature for 15 min and washed three times with PBS buffer. The heart tissue sections were then incubated with WGA staining solution (I3300, Solarbio, China) for 30 min at room temperature in the dark and washed three times with PBS buffer. A fluorescence microscope was used to take fluorescence pictures under an excitation light of 488 nm. ImageJ software was used to measure the cross‐sectional area of individual cell.

### Terminal‐Deoxynucleotidyl Transferase‐Mediated Nick End Labeling (TUNEL) Staining

TUNEL staining was used to assess apoptosis in cardiomyocytes and cardiac tissues using the TUNEL assay kit (A112‐03, Vazyme, China). Pretreated CMs or cardiac tissue sections were fixed with 4% paraformaldehyde for 15 min, and then the samples were permeabilized with Proteinase K and incubated for 20 min at room temperature; 1× equilibration was used to equilibrate the samples, which were incubated for 20 min at room temperature under dark conditions; finally, the samples were incubated with TdT buffer for 30 min at 37 °C. Subsequently, the samples were labeled with α‐actinin (Abcam, UK), at 4 °C overnight followed by Alexa Fluor 594‐conjugated secondary antibody for 1 h. CMs were labeled with α‐actinin (GTX29465, Abcam, UK). Nuclei were labeled with DAPI (C0065; Solarbio, China). Images were captured using a fluorescent microscope (F10Vi, Olympus, Japan) and analyzed with ImageJ software.

### Flow Cytometry Analysis

Apoptosis was quantitatively assessed using the Annexin V‐FITC Apoptosis Detection Kit I (MA‐0220, Meilunbio, China). The manufacturer's instructions were followed. Briefly, primary mouse CMs were processed, digested with EDTA‐free trypsin, and the cell precipitate was collected by centrifugation. The cells were then incubated in Annexin V binding buffer containing Annexin V and PI for 15 min in the dark. All samples were analyzed on a NovoCyteTM II Flow Cytometer (Agilent, China), and data were evaluated using the FlowJo software v. 10.8.1

### Immunofluorescence

The hearts were optimal cutting temperature compound and cut into 4 µm sections. CMs and tissues sections while frozen were fixed with 4% paraformaldehyde. Then, the cells were permeabilized with 0.4% Triton X‐100 in PBS, blocked with goat serum and incubated with primary antibodies against ALYREF (1:200, AB202894, Abcam, UK), γH2AX (1:400, AB81299, Abcam, UK), α‐actinin (1:400, GTX29465, Abcam, UK) at 4 °C overnight followed by Alexa Fluor 488‐or Alexa Fluor (1:400, AB150077, Abcam, UK) 594‐conjugated secondary antibody (1:400, AB150116, Abcam, UK) for 1 h. The cells were counterstained with DAPI (1:50, C0065, Solarbio, China) to label the nuclei. Images were captured using a fluorescent microscope (F10Vi, Olympus, JAPAN) and analyzed with ImageJ software.

### Acute Isolation of Adult Mouse Cardiomyocytes

Adult mouse CMs were acutely isolated as previously described.^[^ ^46^ ^]^ Briefly, mice were anesthetized by intraperitoneal injection of 2% avertin (0.1 mL/10 g) and euthanized by cervical dislocation. The thoracic cavity was opened to expose the heart, and 7 mL of EDTA buffer was slowly injected into the right ventricle using a syringe. The heart was then clipped and transferred to a culture dish containing EDTA buffer. EDTA buffer was injected 2–3 mm above the apical point, and the heart was transferred to a culture dish containing perfusion buffer after slowly perfusing 10 mL of EDTA buffer. Digestion was terminated by continuing to slowly infuse 3 mL of perfusion buffer into the left ventricle and draining the residual EDTA buffer. The hearts were then transferred to a culture dish containing 10 mL of collagenase buffer, and collagenase buffer continued to be slowly injected into the heart tissue until the surface of the heart tissue had an extensively pale and fluffy appearance, indicating that digestion of the heart tissue was complete. The heart tissue was separated into ≈1 mm × 1 mm tissue fragments by blunt separation, and then the enzyme reaction was terminated by adding 5 mL of termination solution. The cell–tissue suspension was filtered through a sterile filter. The cell digest was transferred to a 15 mL centrifuge tube to settle the cells by gravity for 20 min and the supernatant was discarded to collect the cell precipitate. Subsequently, 4 mL of calcium reintroduction buffer was added to the 15 mL centrifuge tube, and a second sedimentation was performed for 10 min to retain the cardiomyocyte precipitate, and the procedure was repeated three times. Cells were resuspended using adherence culture solution and spread into well plates or small dishes at the appropriate density and placed in the cell culture incubator for making them adherent. After 1 h of adherence, the medium was replaced with a new one and the plates were placed in the cell culture incubator for incubation. Under the light microscope, the morphology of the CMs was fully extended, and the cells could be fixed with 4% paraformaldehyde for 20 min at room temperature, followed by immunofluorescence staining and other subsequent steps.

### Statistical Analysis

All the experimental data were expressed as mean ± SEM. The biological replicate (*n*) for each statistical analysis is shown in the figure legends. Shapiro‐Wilk test was used to help determine normality of the data and the assumption of equality of variance. Data sets that conformed to a normal distribution were then analyzed using either the Student's *t*‐test via unpaired two‐tailed Student's *t*‐test, one‐way ANOVA followed with Tukey's post hoc (multigroup analysis). Non‐normally distributed data using Mann–Whitney nonparametric analysis (two‐group analysis) or Kruskal–Wallis nonparametric test with Benjamin–Hochberg method (multigroup analysis). Differences were considered statistically significant at *p* < 0.05.

## Conflict of Interest

The authors declare no conflict of interest.

## Author Contributions

X.G., Y.S., and Z.X., contributed equally to this work. B.C., X.G., B.Y., Z.P., and Y.L. conceived the study concept. X.G., Y.S., Z.X., Z.H., YN. L., and R.G. performed the experiments. X.L., A.C., YN.T., G.L., H.L., X.W., Z.R., N.Z., L.Y., and Y.T. performed or helped interpret and design some key experiments. Z.P., X.G., Z.H., and W.M. wrote and revised the manuscript. B.C., W.M., X.G., and R.G. provided the funding.

## Supporting information



Supporting Information

## Data Availability

The data that support the findings of this study are available from the corresponding author upon reasonable request.
