# Supplementary material for: Preservation of ALYREF Phase Separation Mitigates Doxorubicin‐Induced Cardiomyocyte DNA Damage and Cardiotoxicity
Source: Adv Sci (Weinh). 2025 Sep 3;12(43):e05270. doi: 10.1002/advs.202505270 (PMC12631900; doi:10.1002/advs.202505270)

**Preservation of ALYREF Phase Separation Mitigates Doxorubicin-Induced Cardiomyocyte DNA Damage and Cardiotoxicity**

Xinlu Gao^1,2^*, Yifu Shen^3^*, Zhihui Xiao^1,2*^, Zhenbo Han^4^, Xu Liu^3^, Ao Cai^1,2^, Yanan Tian^1,2^, Guang Lian^1,2^, Wenya Ma^1,2^, Yining Liu^1,2^, Rui Gong^5^, Hanjing Li^1,2^, Xiuxiu Wang^1,2^, Zhongyu Ren^1,2^, Naufal Zagidullin^6^, Lei Yu^7^，Ye Tian^8^, Yu Liu^3#^, Zhenwei Pan^2#^, Baofeng Yang^2#^, Benzhi Cai^1,2,9#^

**Affiliations**

^1^Department of Pharmacy at the Second Affiliated Hospital, Harbin Medical University, Harbin, China

^2^State Key Laboratory of Frigid Zone Cardiovascular Diseases (SKLFZCD), Department of Pharmacology (The State-Province Key Laboratories of Biomedicine-Pharmaceutics of China, Key Laboratory of Cardiovascular Research, Ministry of Education), College of Pharmacy, Harbin Medical University, Harbin, China

^3^Department of Laboratory Medicine at The Fourth Affiliated Hospital, Harbin Medical University, Harbin, China

^4^Department of Pharmacology and Regenerative Medicine, University of Illinois College of Medicine, Chicago, IL 60612, USA

^5^Department of Pharmacy, Xijing Hospital, Fourth Military Medical University, Xi'an, Shanxi, 710032, China.

^6^Department of Internal Diseases, Bashkir State Medical University, Ufa, Russia.

^7^Department of Orthopedic Surgery, The First Affiliated Hospital of Harbin Medical University, Harbin, China.

^8^Department of Pathophysiology and the Key Laboratory of Cardiovascular Pathophysiology, Harbin Medical University, Harbin, China.

^9^Institute of Clinical Pharmacy, NHC Key Laboratory of Cell Transplantation, the Heilongjiang Key Laboratory of Drug Research, Harbin Medical University, Harbin, China

*These authors contributed equally to this work.

**^#^Corresponding authors:**

Prof. Benzhi Cai, Department of Pharmacy at the Second Affiliated Hospital, Harbin Medical University, E-mail: caibz@ems.hrbmu.edu.cn;

Prof. Baofeng Yang, State Key Laboratory of Frigid Zone Cardiovascular Diseases (SKLFZCD), Department of Pharmacology (The State-Province Key Laboratories of Biomedicine-Pharmaceutics of China, Key Laboratory of Cardiovascular Research, Ministry of Education), College of Pharmacy, Harbin Medical University, E-mail: yangbf@ems.hrbmu.edu.cn;

Prof. Yu Liu, Department of Laboratory Medicine at The Fourth Affiliated Hospital, Harbin Medical University, E-mail: liuyuhmu@hrbmu.edu.cn;

Prof. Zhenwei Pan, PhD, Department of Pharmacology, State-Province Key Laboratories of Biomedicine-Pharmaceutics of China, Harbin Medical University, Email: panzw@ems.hrbmu.edu.cn

**
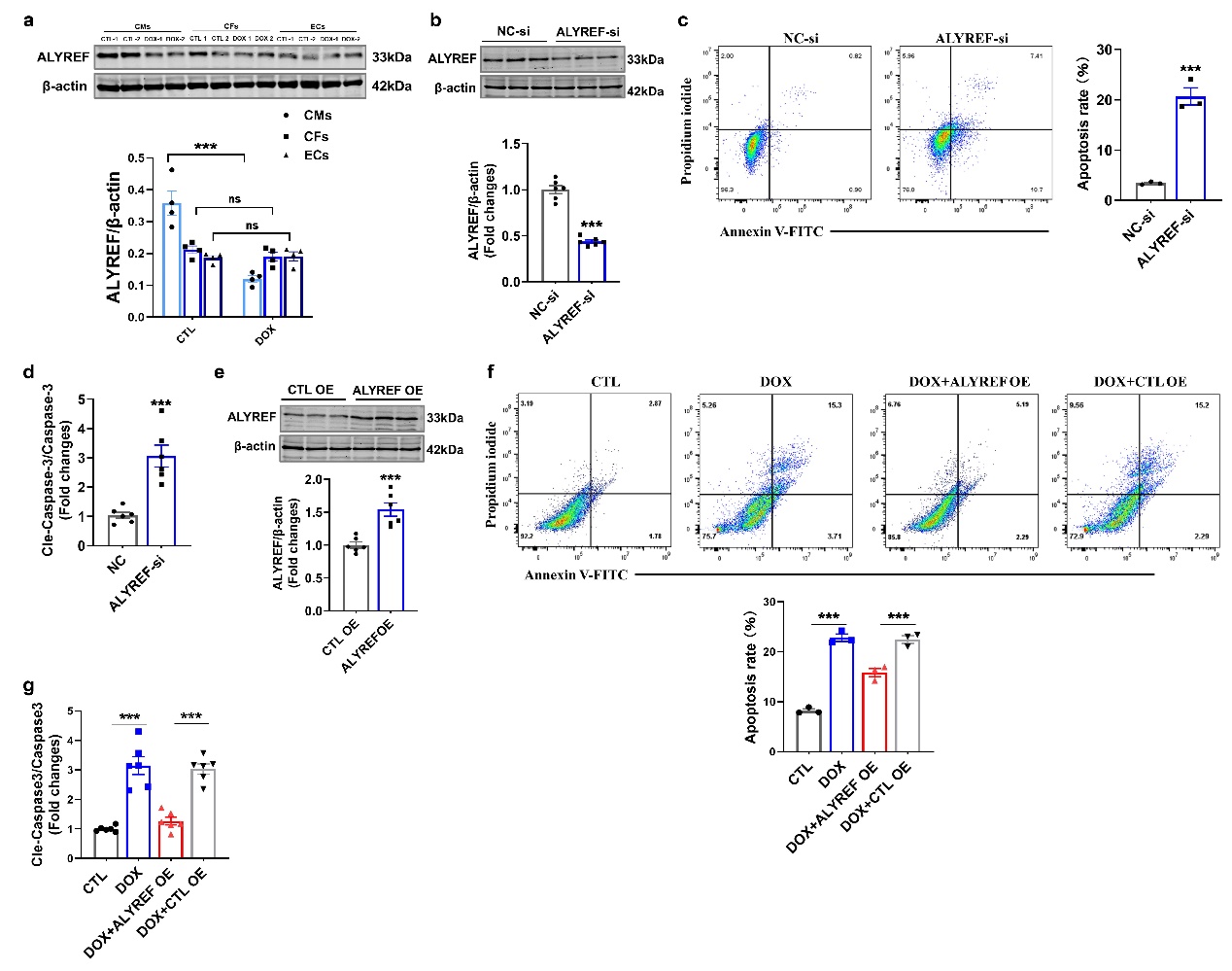
**

**Figure S1. ALYREF regulates DNA damage and apoptosis in cardiomyocytes**

**a)** Western blot analysis of ALYREF protein levels in DOX-treated neonatal mouse CMs, myocardial fibroblasts or endothelial cells (*n* = 4). **b)** Western blot analysis of ALYREF protein levels in neonatal mouse CMs after transfected with ALYREF siRNA (*n* = 6). **c)** Representative flow cytometry images of Annexin V-FITC/PI staining in CMs after transfection with ALYREF siRNA (*n* = 3). **d)** Cle-Caspase-3 to Caspase-3 ratio statistics. **e)** Western blot analysis of ALYREF protein levels in CMs after transfected with ALYREF plasmid (*n* = 6). **f)** Representative flow cytometry images of Annexin V-FITC/PI staining in CMs after transfected with ALYREF (*n* = 3). **g)** Cle-caspase3 to Caspase3 ratio statistics. Data are presented as mean ±SEM. Statistical analysis was performed by Student t test for **a-c,** **e, f**. one-way ANOVA with Tukey’s multiple comparisons test was used in **h.** ^***^*P* < 0.001.


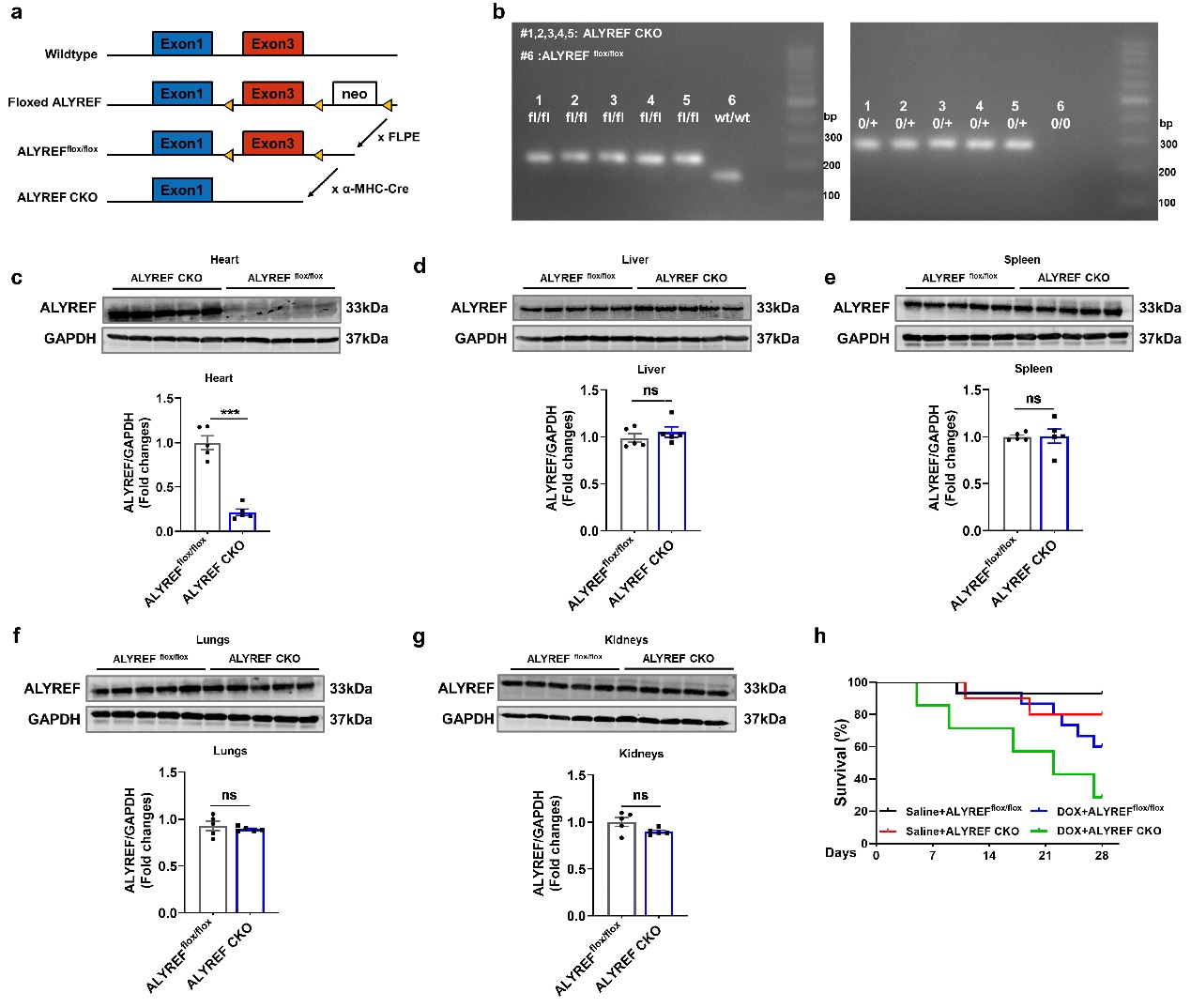


**Figure S2. Cardiac-specific ALYREF deficiency exacerbates Doxorubicin induced cardiotoxicity in mice**

**a)** Schematic diagram showing the breeding strategy. After the introduction of cre-recombinase (α-MHC-Cre), the ALYREF gene Exon 3 was specifically excised in CMs, resulting in generation of selective ALYREF knockout mice (ALYREF CKO)**. b)** Representative genotyping results for ALYREF CKO and control littermates. Pups 1-5 were identified as ALYREF CKO [ALYREF^fl/fl^ with cre recombinase (Cre0/+)], and pups 6 was control littermates [ALYREF ^fl/fl^ without cre recombinase (Cre0/0)]. **c-g)** Western blot analysis of ALYREF expression levels in mice heart, liver, spleen, lungs, kidneys (*n* = 5). **h)** Kaplan-Meier survival curves of mice in each group (*n* = 15, 12, 15, 12 mice per group). Data are presented as mean ±SEM. Statistical analysis was performed by Student t test for **c** through **g**. ^***^*P* < 0.001.


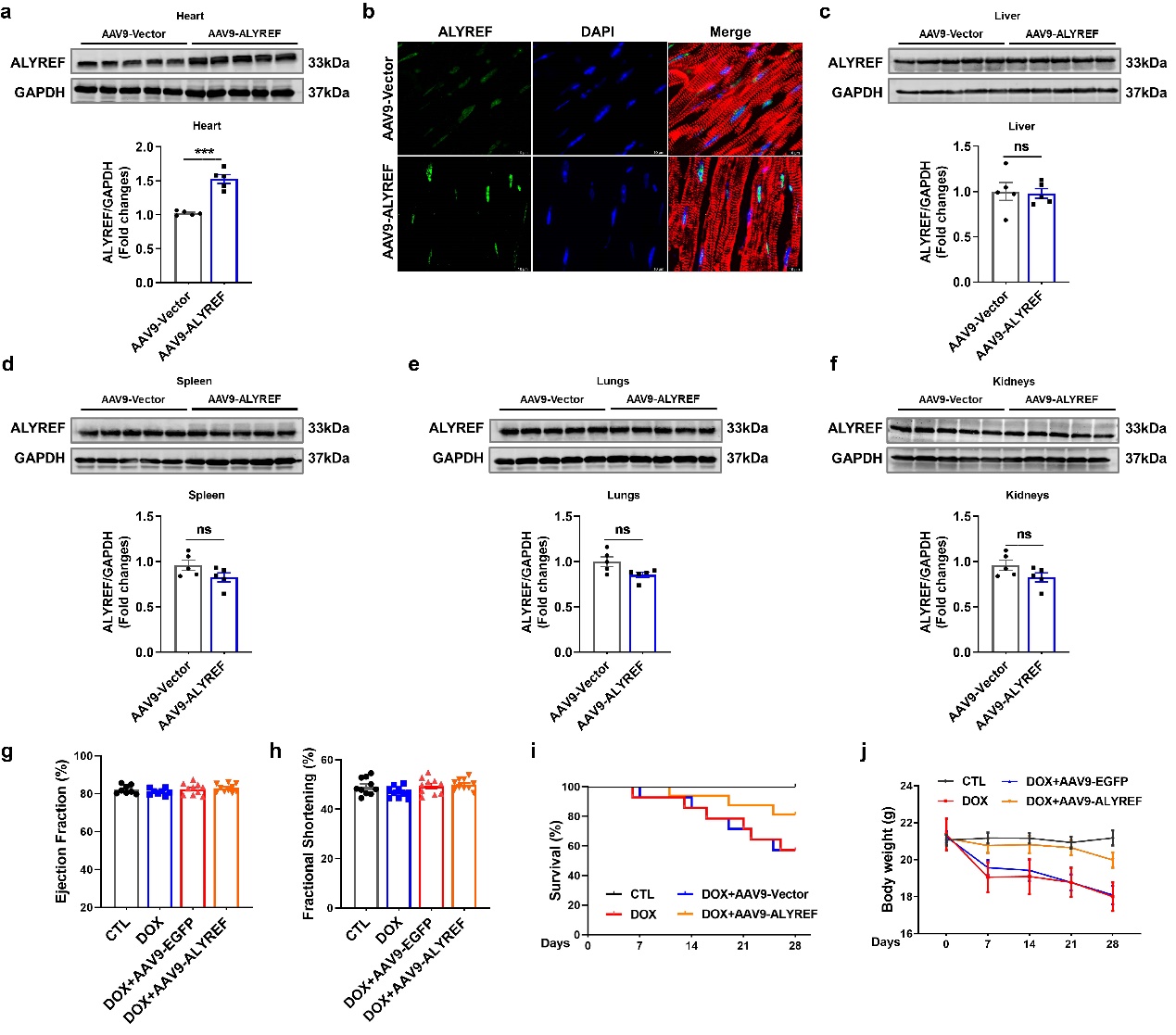


**Figure S3. Cardiac-specific overexpression of ALYREF attenuates Doxorubicin-induced cardiotoxicity**

**a, c-f)** Western blot analysis of ALYREF expression levels in mice heart, liver, spleen, lungs, kidneys (*n* = 5). **b)** Representative images of immunostaining of ALYREF (green) in heart (*n* = 3). Cardiac tissue was counterstained with α-actinin (red, a cardiac marker) and DAPI (blue). Scale bar = 50 μm. **g, h)** Cardiac function of basal levels was examined by echocardiography in groups of mice (*n* = 10). **i)** Kaplan-Meier survival curves of mice in each group (n=15, 12, 15, 12 mice per group). **j)** Body weight analysis of mice in each group (*n* = 10). Data are presented as mean±SEM. Statistical analysis was performed by Student t test for **a, c-f**. Statistical analysis was performed by one-way ANOVA with Tukey’s multiple comparisons test for **g, h**. ^***^*P* < 0.001.


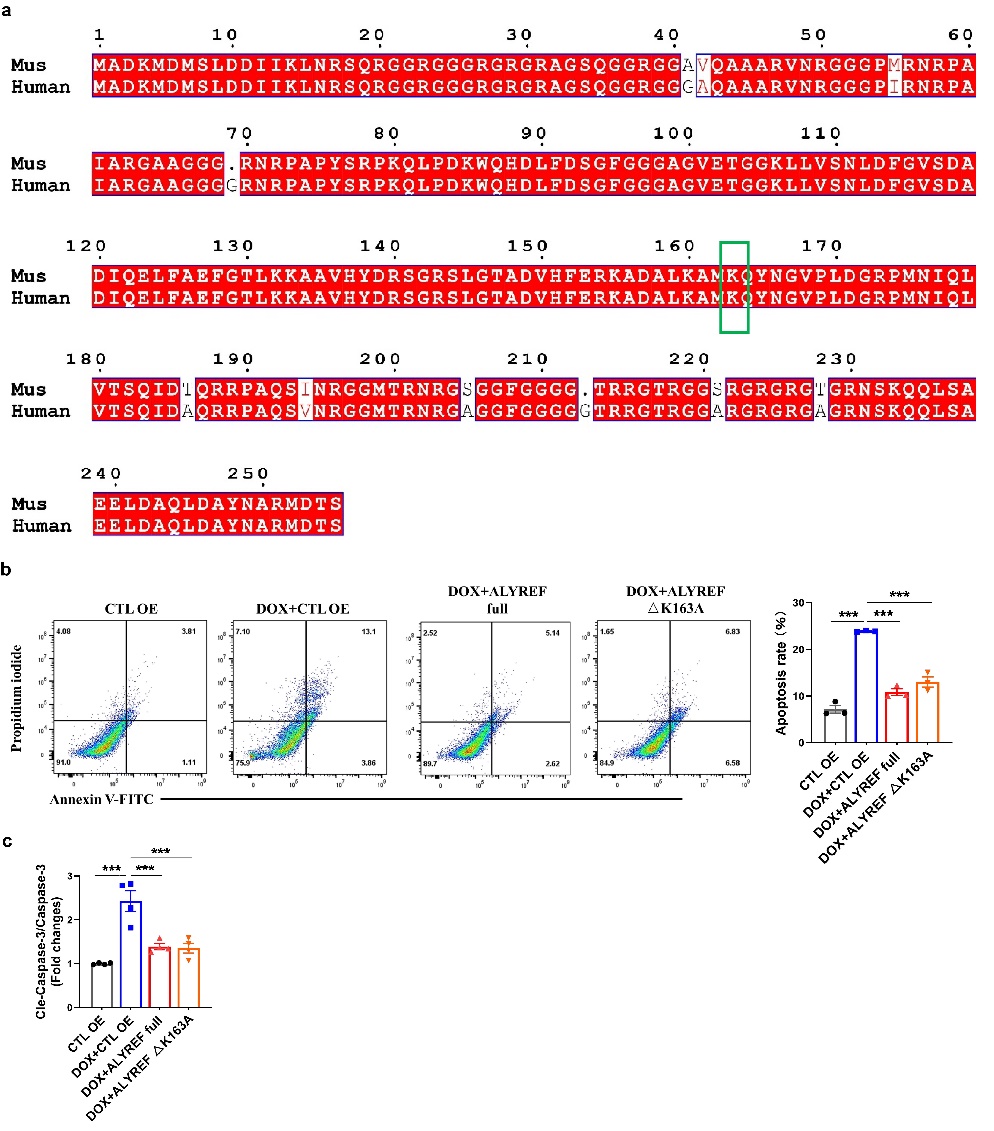


**Figure S4. ALYREF regulates doxorubicin-induced DNA damage and apoptosis in cardiomyocytes independent of m^5^C reader function**

**a)** Human and mouse ALYREF protein sequence comparison. **b)** Representative flow cytometry images of Annexin V-FITC/PI staining in CMs after transfected with ALYREF or ALYREF △K163A plasmid and then treated with DOX. **c)** Cle-Caspase3 to Caspase3 ratio statistics. Data are presented as mean±SEM. one-way ANOVA with Tukey’s multiple comparisons test was used in **b** and **c.** ^**^*P* < 0.01 and ^***^*P* < 0.001.


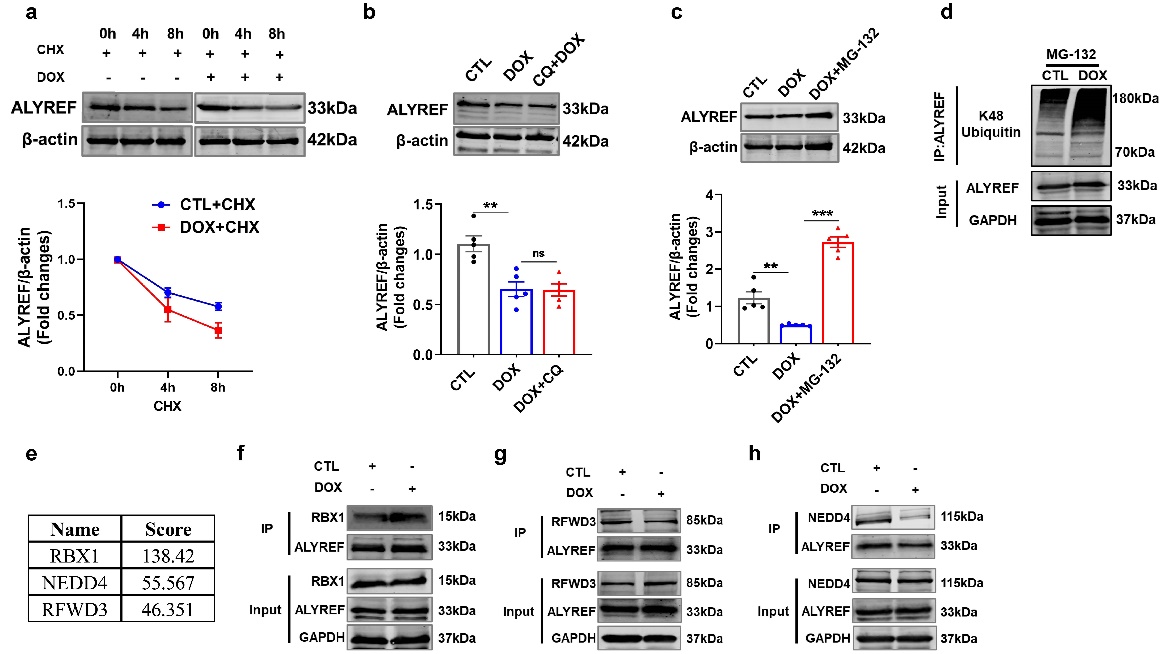


**Figure S5.** **Doxorubicin induces ubiquitination degradation of ALYREF protein**

**a-c）**Western blot analysis of ALYREF protein levels in CMs after cotreated with DOX and the protein synthesis inhibitor CHX, the autophagy lysosome inhibitor Chloroquine (CQ), and the protease inhibitor MG-132 (*n* = 5). **d)** Immunoprecipitation for detection of ALYREF ubiquitination level at K48 position in CMs after treated with MG-132 and DOX. **e)** Mass spectrometry analysis of E3 ubiquitin ligase bound to ALYREF. **f-h)** Immunoprecipitation analysis of ALYREF binding to E3 ubiquitin ligase in CMs treated with DOX (*n* = 3). Data are presented as mean±SEM. Statistical analysis was performed by one-way ANOVA with Tukey’s multiple comparisons test for **b** and **c**. ^**^*P* < 0.01, and ^***^*P* < 0.001.


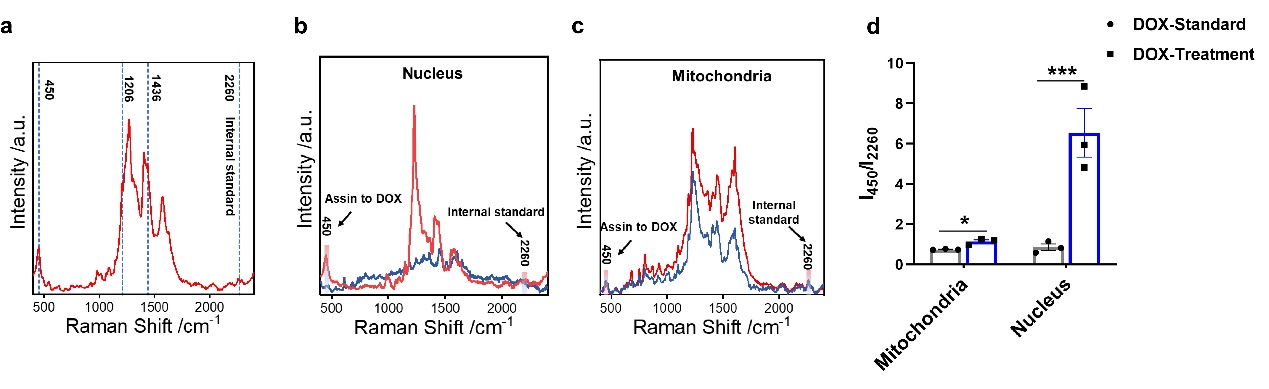


**Figure S6. Quantitative analysis of doxorubicin in cardiomyocyte organelles**

**a)** the Surface enhanced Raman spectroscopy (SERS) spectra of DOX. **b, c)** SERS spectra of DOX were extracted from isolated CMs nuclei and mitochondria in CMs treated with DOX. Red line: fingerprints of DOX-treated CMs in nuclei or mitochondria; blue line: fingerprints of DOX standard in nuclei or mitochondria. **d)** Relative peak intensities of DOX in the nucleus or mitochondria of CMs at characteristic peaks 450 and 2260 cm^-1^. Data are presented as mean±SEM. one-way ANOVA with Tukey’s multiple comparisons test was used in **d**. ^*^*P* < 0.05 and ^***^*P* < 0.001.


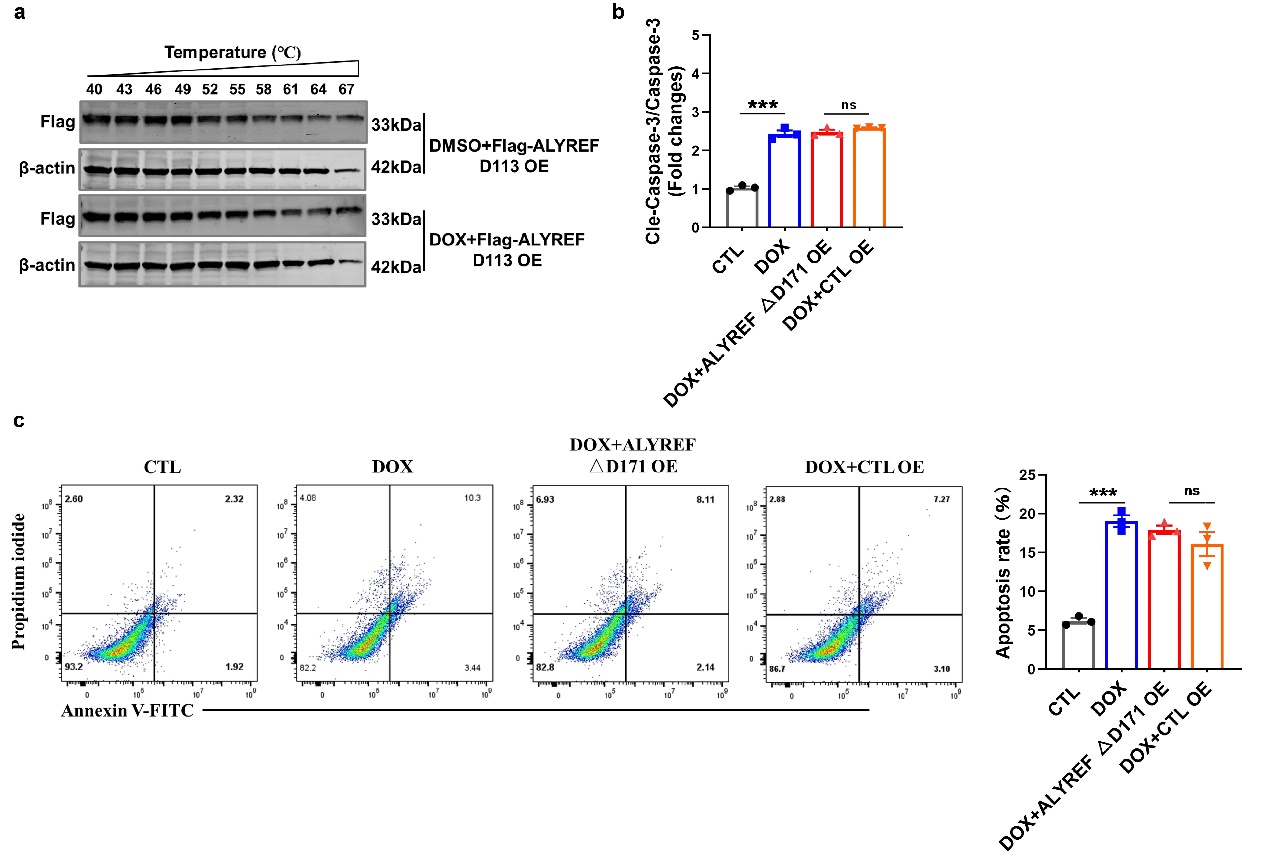


**Figure S7.** **Doxorubicin is not bound to the D131^st^ amino acid of ALYREF.**

**a)** Representative images of CESTA of Flag-ALYREF △D131 in CMs treated with DOX. CMs were transfected by Flag-tagged mutant plasmids with D131 deletion in ALYREF (ALYREF D131). **b)** Cle-Caspase3 to Caspase3 ratio statistics. **c)** Representative flow cytometry images of Annexin V-FITC/PI staining in CMs after transfected with ALYREF△D171 and then treated with DOX. Data are presented as mean±SEM. Statistical analysis was performed by one-way ANOVA with Tukey’s multiple comparisons test for **b**. ^***^*P* < 0.001.


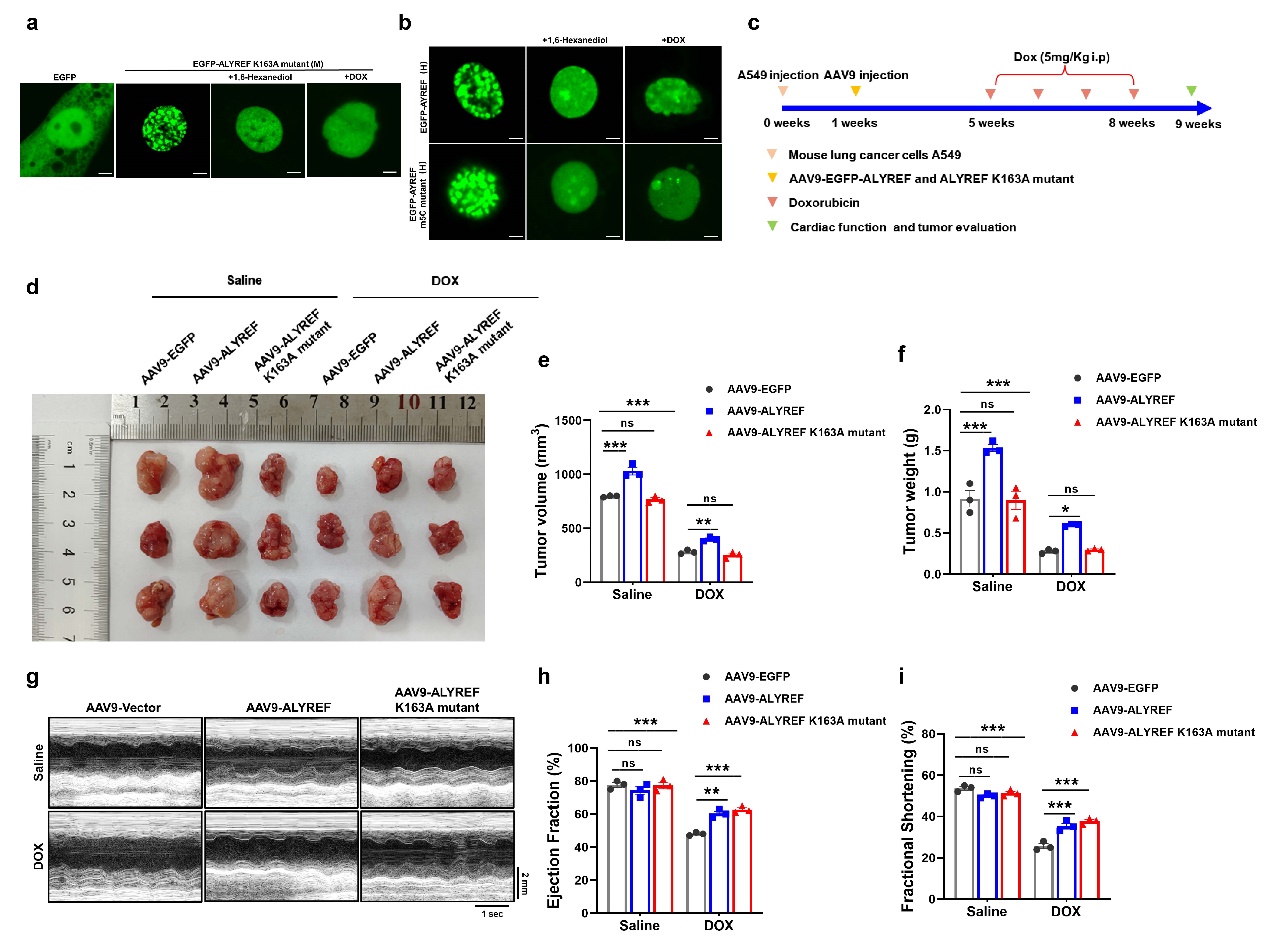


**Figure S8. Effect of ALYREF on tumor proliferation is independent of its phase separation ability**

**a**) Representative images of the CMs overexpressing either EGFP, EGFP- ALYREF K163A mutant (Mouse, M). Scale bars=10 μm. **b)** Representative images of the HEK-293T cells overexpressing either EGFP-ALYREF (Human, H) or EGFP-ALYREF m^5^C mutant (H) and then treated with 1,6-HEX. Scale bars =10 μm. **c)** Schematic diagram of animal experiment process. **d)** Representative anatomical images of tumor size. scale bar = 1 cm in each group of mice (*n* = 3). **e)** Comparison of tumor volume among each group of mice (*n* = 3). **f)** Comparison of tumor weight among each group of mice (*n* = 3). **g)** Cardiac function was examined by echocardiography in treating DOX or saline mice after overexpression of AAV9-Vector, AAV9-ALYREF and AAV9- ALYREF K163A mutant. **h, i)** Comparison of EF and FS among each group of mice (*n* = 3). Dates are presented as mean ±SEM. Statistical analysis by one-way ANOVA with Tukey’s multiple comparisons test for **e, f, h,** and **i**. ^*^*P* < 0.05, ^**^*P* < 0.01, ^***^*P* < 0.001.


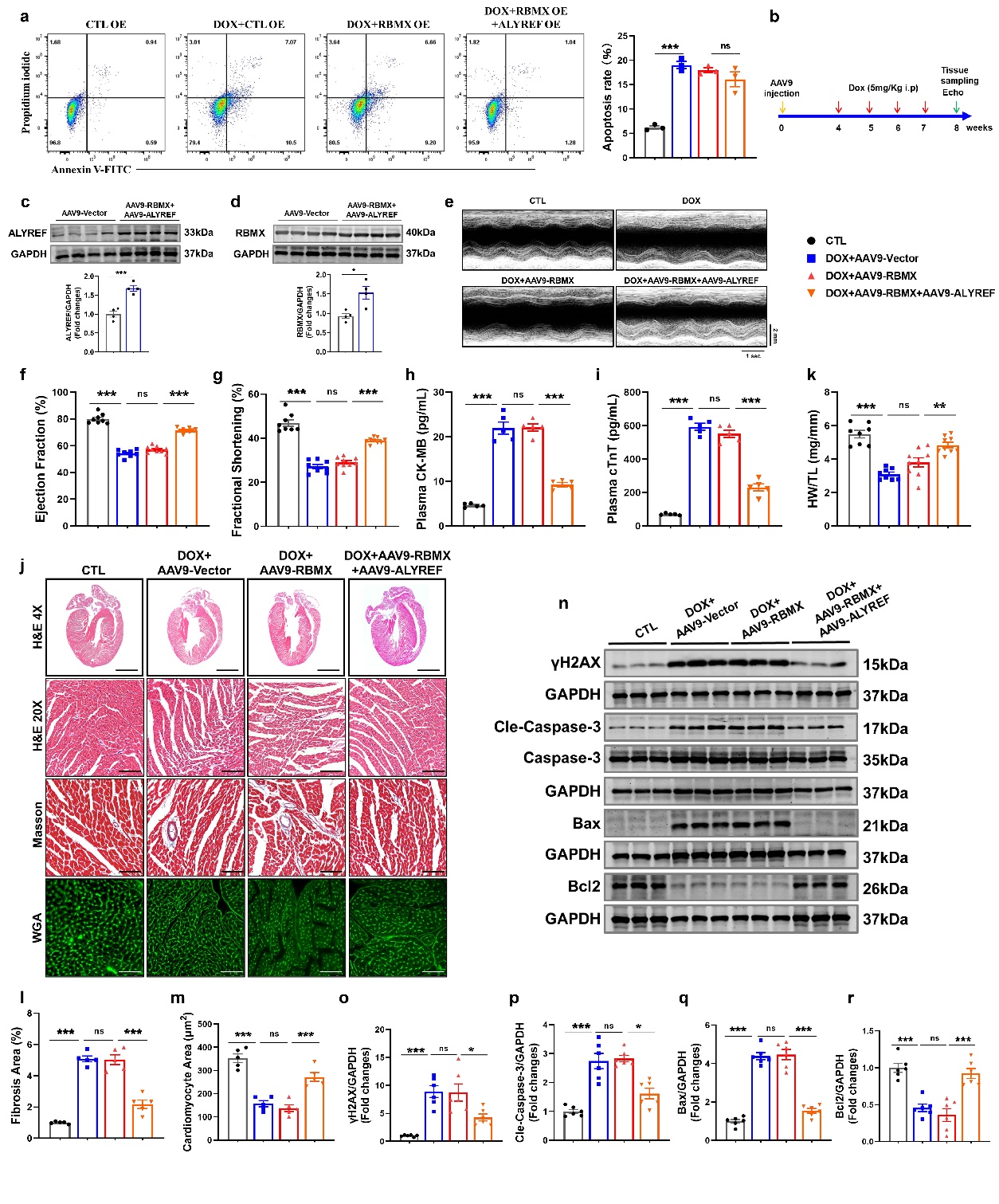


**Figure S9. Doxorubicin affects genome stability by causing dissociation of the NRAC1 complex in cardiomyocytes**

**a)** Representative flow cytometry images of Annexin V-FITC/PI staining in CMs after co-transfection with RBMX and ALYREF plasmid and then treated with DOX (*n* = 3). **b)** Schematic diagram of animal experiment process. **c, d)** Western blot analysis of ALYREF and RBMX expression levels in mice heart (*n* = 4). **c-e)** Cardiac function was examined by echocardiography of mice in each group. Quantitative analysis of ejection fraction (EF), shortening fraction (FS), end-diastolic left ventricular internal diameter (LVIDd), and end-systolic left ventricular internal diameter (LVIDs) (*n* = 8). **f, g)** ELISA to measure creatine kinase-MB (CK-MB) and cardiac troponin T(cTnT) levels in the serum (*n* = 5). **h)** Representative hematoxylin and eosin (H&E), Masson, Sirius red and WGA staining of heart (*n* = 5). **i)** Heart weight to tibia length (HW/TL) ratio (*n* = 5). **j, k)** Quantitative analysis of Masson and WGA staining (*n* = 5). **l-p)** Western blot analysis of γH2AX (*n* = 6), Cleaved-Caspase3 (*n* = 6), Bax (*n* = 6), and Bcl2 (*n*= 6) protein levels in heart. Data are presented as mean ± SEM. one-way ANOVA with Tukey’s multiple comparisons test was used in **d** through **g**, and **i** through **l**. ^*^*P* < 0.05, ^**^*P* < 0.01 and ^***^*P* < 0.001.

**Graphic Abstract**


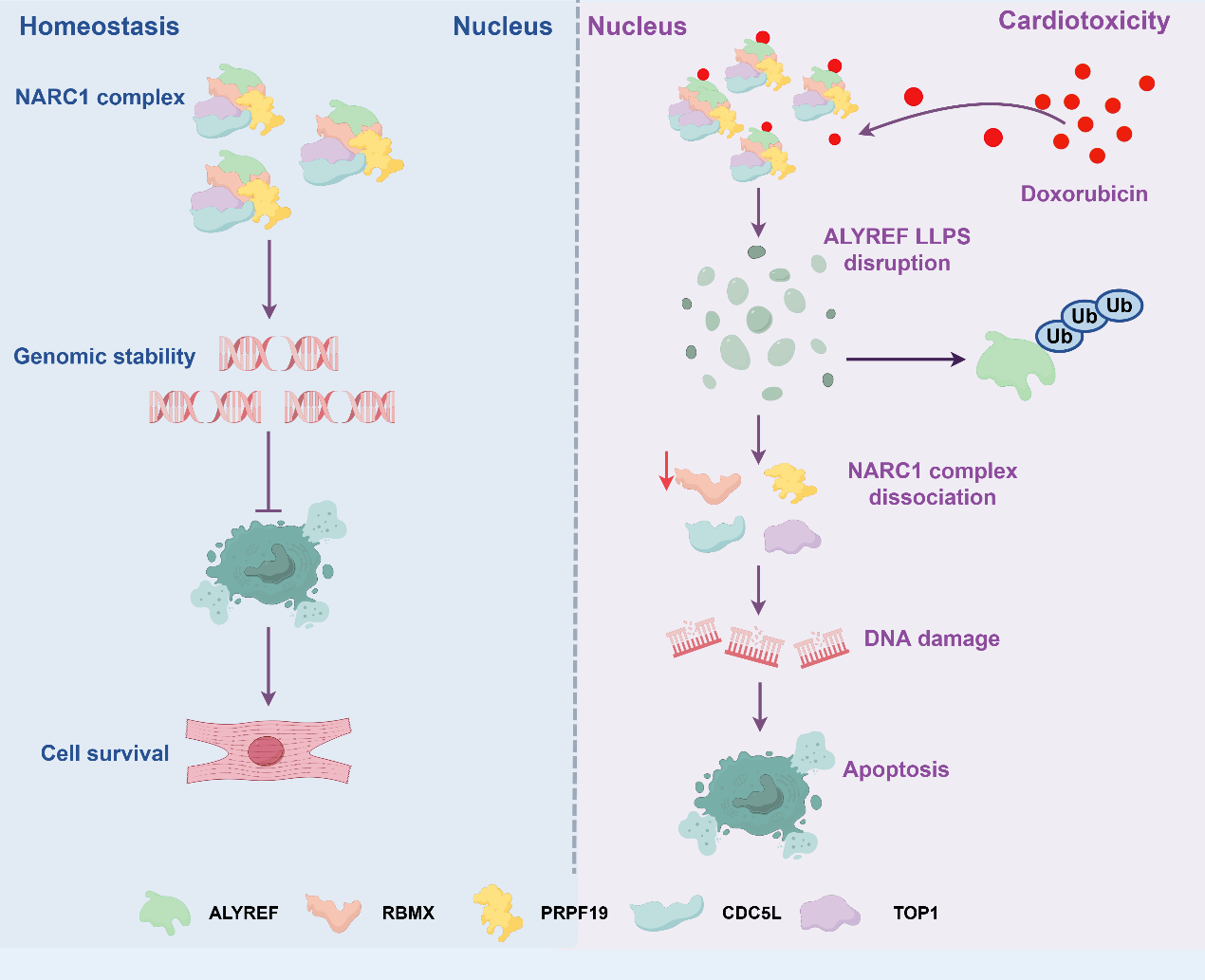

Supplement: Supplementary file 1 — Supporting Information [file ADVS-12-e05270-s001.docx]
